# Current Developments in Antibacterial Nanoparticle–Based Dental Composite Materials: A Comprehensive Overview

**DOI:** 10.1155/ijod/7065782

**Published:** 2026-09-24

**Authors:** Athulya Mullappally, Vishnu Vijay Kumar, Arpitha G R

**Affiliations:** ^1^ Manipal Institute of Technology Bengaluru, Manipal Academy of Higher Education, Manipal, India, manipal.edu; ^2^ Structural Engineering, Division of Engineering, New York University Abu Dhabi (NYUAD), P.O. Box 129188, Abu Dhabi, UAE, nyu.edu; ^3^ Faculty of Engineering, Higher Colleges of Technology, Abu Dhabi 25035, UAE, hct.ac.ae

**Keywords:** antibacterial, composite, dental, dental caries, nanoparticle, remineralization

## Abstract

The persistence of secondary caries, biofilm accumulation, and hybrid layer degradation as leading causes of failure in resin‐based composite (RBC) restorations underscores the urgent need for materials that can effectively prevent demineralization and promote the remineralization of dental hard tissues. Conventional composites are primarily bioinert, offer minimal defense against cariogenic biofilms, and lack the ability to restore mineral loss at the tooth–restoration interface. Recent advancements in nanotechnology have paved the way for the development of antibacterial and bioactive composites that integrate metallic, metal–oxide, polymeric, organic, and ion‐releasing nanoparticles (NPs). These innovative systems provide antimicrobial effects through contact or ion‐mediated mechanisms, enhance the stability of the hybrid layer, and increasingly demonstrate potential for remineralization through the controlled release of calcium, phosphate, or fluoride ions. This review provides a critical analysis of the current antibacterial bioactive resin–based nanocomposites employed in dentistry, covering the PubMed, Scopus, Web of Science, and Google Scholar databases with keywords silver (Ag), zinc oxide, titanium dioxide, and magnesium oxide NPs (MgO‐NPs); quaternary ammonium polyethylenimine (QA‐PEI)–based systems; chitosan‐derived NPs; and remineralization‐promoting fillers such as bioactive glass (BG) and primarily covering studies published between 2020 and 2026. Key challenges, including NP agglomeration, aesthetic modifications, variable biocompatibility, uncontrolled ion release, and the absence of standardized evaluation protocols, are examined in relation to their impact on predictable remineralization and clinical outcomes. Most current evidence remains preclinical, with limited in situ and early translational validation. This comprehensive narrative review included studies investigating NP‐modified dental restorative materials and excluded duplicate records, nondental applications, and studies not relevant to restorative dentistry.

## 1. Introduction

Resin‐based composites (RBCs) have gained prominence as the preferred material for direct dental restorations. This is largely attributed to their ability to facilitate minimally invasive cavity preparation, form micromechanical bonds with tooth structures, and deliver exceptional aesthetic results. However, their long‐term effectiveness is often undermined by the development of secondary cavities and fractures at the bulk or margins. Numerous surveys and retrospective studies have consistently identified secondary caries as the most prevalent cause of composite restoration replacement, accounting for 40%–60% of failures, depending on the cohort and duration of observation [1–3]. Recent systematic reviews and large‐scale clinical evaluations have consistently identified secondary caries and restoration fracture as the leading causes of failure in RBC restorations. The relative contribution of secondary caries varies according to patient risk profile, restoration type, and follow‐up duration; however, contemporary evidence indicates that biofilm‐mediated degradation at restoration margins remains a major factor limiting the longevity of direct composite restorations. The etiological principle underlying secondary caries is the formation of cariogenic biofilms at or near restoration margins. These biofilms, typically dominated by *Streptococcus mutans* and lactobacilli, produce organic acids that diffuse into tooth tissues, demineralizing enamel and dentin [4]. Because conventional RBCs are essentially bioinert, they do not actively suppress bacterial colonization or neutralize acidic microenvironments. Furthermore, polymerization shrinkage and hydrolytic or enzymatic degradation of the hybrid layer can create microgaps that favor bacterial ingress and nutrient diffusion, thereby exacerbating the risk of interfacial demineralization [5]. Over the past two decades, there has been an intense research effort to engineer antibacterial dental resins, relying initially on soluble agents such as chlorhexidine or silver (Ag) salts, then covalently bound antibacterial monomers such as quaternary ammonium compounds (QACs), and more recently, on nanostructured fillers that introduce antibacterial and bioactive functions without seriously compromising mechanical performance [6–9]. The integration of nanoparticles (NPs) into composite formulations can, in principle, address several intrinsic weaknesses of conventional RBCs [10]. Nanoscale fillers possess a significantly high surface area, enhancing their interactions with microorganisms and the surrounding fluids. Nanoscale metallic and metal oxide fillers can release antibacterial ions and generate reactive oxygen species (ROS), providing effective contact‐based antimicrobial activity with minimal leaching. Additionally, bioactive glass (BG) NPs can release calcium, phosphate, fluoride, and other beneficial ions, thereby promoting remineralization and stabilization of the hybrid layer [11–14]. This review examines antibacterial bioactive dental composites that incorporate NPs, which confer both antibacterial properties and a degree of bioactivity, such as pH buffering, ion release, or remineralization.

The aim of this review is to critically summarize current developments in antibacterial NP–based dental composites, focusing on their antibacterial mechanisms, bioactive functions, material properties, evaluation methods, limitations, and future clinical potential. A literature review was executed using databases such as PubMed, Scopus, Web of Science, and Google Scholar, concentrating on publications from 2020 to 2026. The search incorporated terms such as Ag, zinc oxide, titanium dioxide, and magnesium oxide NPs (MgO‐NPs); systems based on quaternary ammonium polyethylenimine (QA‐PEI); chitosan‐derived NPs; and fillers that promote remineralization, including BG. Both original research and review articles that explored the antimicrobial, mechanical, physicochemical, biocompatibility, ion‐release, remineralization, and clinical aspects of these materials were included. Exclusion criteria were applied to studies not directly associated with resin‐based dental composites or their antibacterial and bioactive uses. The assessment of predictable remineralization and clinical performance accounted for significant translational challenges, including NP agglomeration, aesthetic alterations, inconsistent biocompatibility, uncontrolled ion release, and the lack of standardized evaluation protocols. Current methods for evaluating antibacterial performance and bioactivity are discussed, along with key limitations related to biocompatibility, aesthetics, NP dispersion, ion release, and long‐term clinical applicability. The review highlights existing knowledge gaps and future research directions required to facilitate the clinical translation of multifunctional NP–enhanced dental restorative materials. Evidence regarding long‐term clinical effectiveness, durability, patient outcomes, and safety remains limited. Consequently, many proposed antibacterial and remineralization mechanisms should be regarded as promising biological rationales rather than clinically established therapeutic outcomes.

## 2. Secondary Caries, Biofilms, and the Rationale for Antibacterial Bioactive Dental Composites

Dental caries is best understood as a biofilm‐mediated yet multifactorial disease arising from the dynamic interplay between microbial activity, diet, host defenses, and environmental influences [15, 16]. While dysbiosis within the dental biofilm plays a central role, the development of caries is also strongly influenced by frequent consumption of fermentable carbohydrates, particularly sugars, which promote the proliferation of acidogenic and aciduric microorganisms such as *S. mutans* and *Lactobacillus* spp. [17]. Moreover, host‐related factors, including saliva flow rate, buffering capacity, and tooth morphology, significantly influence caries susceptibility by modulating the balance between demineralization and remineralization processes [18]. Behavioral and environmental factors such as oral hygiene practices, fluoride exposure, and genetic predisposition further contribute to disease progression [19]. These acids diffuse into the enamel and dentin, dissolve hydroxyapatite (HA), and lead to subsurface demineralization [4, 20]. The replacement of localized lost tooth structure with composite restorations can establish an ecological niche at the restoration–tooth interface, which may facilitate microbial adhesion and colonization. To a great extent, this problem is explained by the aspects of materials and methods applied in the case of polymerization shrinkage of resin composites. This contraction is capable of undermining the marginal seal and causing microscopic interfaces between the tooth and the restoration. Such defects can allow entry of oral fluids, bacteria, and metabolic by‐products, hence facilitating the development of microleakage and certain biofilms around the borders of the restoration [21]. Fissures, marginal defects, and surface irregularities in restorative materials can increase plaque retention and create hidden environments that support the growth of cariogenic bacteria [22]. These conditions are particularly significant in the development of secondary caries, which often occur at the junctions of existing restorations where the restoration meets the cavity, and biofilm activity can cause localized demineralization of adjacent dental tissues [23, 24]. Surface roughness, marginal gaps, and degraded adhesive interfaces favor plaque retention, whereas the absence of any intrinsic antibacterial activity in most commercially available composites allows biofilms to thrive [25, 26]. Recurrent caries typically manifests at the gingival margin of Class II restorations and at cervical areas of Class V or root‐surface restorations, where plaque removal is more difficult [27]. Traditional strategies for caries prevention, such as the use of fluoride‐releasing liners, maintaining optimal oral hygiene, and providing dietary counseling, are crucial. However, these approaches do not fully mitigate the susceptibility of resin–dentin interfaces and restoration surfaces. In addition to these preventive measures, the surface properties of restorative materials are pivotal in influencing bacterial adhesion and biofilm development [28]. Surface finishing and polishing techniques are routinely applied to minimize the surface roughness of resin composites, which is a key factor in plaque accumulation [29]. Empirical studies have shown that when surface roughness exceeds a critical threshold of ~0.2 µm, there is a significant increase in bacterial adhesion and plaque retention [30, 31]. This underscores the necessity of employing effective finishing and polishing methods to limit biofilm formation on restorative materials. The choice of polishing systems and surface treatments can modify the topography of composite restorations, thereby affecting microbial colonization and the durability of restorations over time [32]. Therefore, it seems rational to design restorative materials that can inhibit biofilm formation and support remineralization at their margins, especially in high‐risk patients [33, 34]. The concept of bioactive composite emerged from the success of glass ionomer cements (GICs) and BGs in stimulating apatite formation and releasing fluoride and other ions [35, 36]. When such bioactive fillers are combined with antibacterial NPs, the result can be a multifunctional nanocomposite capable of suppressing bacterial growth, elevating local pH, releasing remineralizing ions, and inhibiting host‐derived matrix metalloproteinases (MMPs) within demineralized dentin [34, 37, 38]. While inhibiting bacterial growth or biofilm formation is often suggested as a means of reducing the risk of secondary caries, there are important limitations to consider. Laboratory‐based bacterial inhibition does not always translate to caries prevention in the clinic. Secondary caries is a multifactorial process influenced by microbial ecology, dietary habits, saliva composition, oral hygiene, restoration quality, and host‐related factors. Therefore, in vitro antibacterial activity should be considered as a surrogate marker of the therapeutic activity but not necessarily a marker of clinical effectiveness.

## 3. Antibacterial Strategies in Resin‐Based Materials and the Role of NPs

Antibacterial strategies for RBCs can be broadly classified into release‐based, contact‐active, and NP‐based approaches [33, 34]. Release‐based systems incorporate soluble agents such as chlorhexidine, fluoride salts, antibiotics, or Ag salts into the resin matrix or filler particles [39]. These materials can produce a clear inhibition zone in agar diffusion tests but often show an initial burst release followed by rapid depletion, potentially compromising mechanical properties and raising concerns about long‐term cytotoxicity and resistance [34, 40]. Contact‐active systems use cationic antibacterial monomers (like methacryloyloxydodecylpyridinium bromide, dimethylaminododecyl methacrylate [DMADDM], and dimethylaminohexadecyl methacrylate [DMAHDM]) that are covalently bonded to the polymer network [41–43]. It is important to distinguish polymerizable quaternary ammonium monomers, such as DMADDM and DMAHDM, from QA‐PEI NPs. Polymerizable QACs are incorporated directly into the resin network through copolymerization and provide antibacterial activity via permanently charged resin surfaces. In contrast, QA‐PEI consists of cross‐linked NPs dispersed within the material, acting as particulate contact‐active antibacterial agents. These quaternary ammonium monomers present a positively charged surface that disrupts bacterial membranes upon contact while minimizing leaching [44]. NP‐based strategies may rely on drug release, contact killing, or a combination of both [45]. Metallic NPs such as Ag can release Ag^+^ ions with broad‐spectrum antimicrobial activity; metal oxide NPs such as ZnO, TiO_2_, and MgO can generate ROS and release inhibitory cations; and polymeric NPs such as QA‐PEI provide highly efficient contact‐killing surfaces [13, 46, 47]. In addition, BG, surface prereacted glass (S‐PRG), and calcium phosphate (CaP) NPs release remineralizing ions and may indirectly affect the microbial ecology by increasing local pH or supplying fluoride [35, 36, 48, 49]. In many contemporary formulations, polymerizable QAC monomers are combined with antibacterial and bioactive NPs to achieve synergistic effects and mitigate the drawbacks associated with any single strategy [50, 51]. It should be clarified that the evidence presented in this review comes from various dental materials such as dental restorative composites, dental adhesive systems, orthodontic materials, dental prosthetic materials, and dental materials used with implants. The conclusions from restorative composites and adhesives are most specific to the clinical aspect of this review. The observations made in orthodontic, prosthetic, and implant‐related studies are mostly meant for providing mechanistic information, antibacterial information, or biocompatibility information and cannot be interpreted as evidence of performance in the context of restorative composite applications. However, it should be noted that there are many testing strategies for determining the presence of an antibacterial agent that are not necessarily interchangeable due to different biological endpoints being assessed. Agar diffusion tests focus mainly on the diffusion of antimicrobial agents through a medium and will underestimate the activity of nonleaching and/or contact‐active materials. Direct contact tests measure the antibacterial activity at the surface of the material and are appropriate for immobilized antibacterial systems. Colony‐forming unit (CFU) assays measure viable microorganisms but do not shed light on metabolic activity or architecture of the biofilm. Bacterial viability and activity are assessed by metabolic assays, and this is not necessarily a direct correlation with viable counts. However, multispecies biofilm models more closely approximate the complexity of the oral cavity and are more variable to run experiments. Thus, variations in antibacterial performance across studies could be due to methodological variations and not necessarily to actual differences in material performance, and caution should be taken to interpret and compare the results.

## 4. Metallic NPs in Antibacterial Bioactive Dental Composites

### 4.1. Ag NPs (AgNPs)

Ag has long been recognized as a potent antimicrobial metal with activity against Gram‐positive and Gram‐negative bacteria, fungi, and viruses [52]. In the nanostructured form, AgNPs have a very large surface area and can release substantial amounts of Ag^+^ ions, even at low filler loadings [53]. Ag^+^ interacts with bacterial cell walls and membranes, binds the thiol groups of enzymes, interferes with DNA replication, and induces ROS formation, leading to cell death [54]. AgNPs have been directly incorporated into experimental and commercial composite resins, flowable materials, and adhesives. Early studies showed that adding as little as 0.5–1 wt% AgNPs to composite resins significantly reduced *S. mutans* viability and lactic acid production without severely compromising flexural strength or hardness, although the extent of color change varied with NP size and dispersion [25, 55, 56]. More sophisticated approaches embed Ag within BG networks. Chatzistavrou et al. [11] synthesized a sol–gel–derived Ag‐doped BG (Ag‐BG) and incorporated it into a flowable resin composite at 1–15 wt%. They demonstrated pronounced bactericidal activity against oral streptococci, the formation of a hydroxycarbonate apatite layer after immersion in simulated body fluid (SBF), and the preservation of bond strength to dentin, particularly at intermediate filler levels [12]. Subsequent investigations have corroborated that these Ag‐BG–containing composites exhibit prolonged Ag‐ion release, alkalinization of the surrounding environment, and sustained inhibition of multispecies biofilms while preserving flexural strength and modulus within clinically acceptable parameters [11, 13]. The Ag‐BG composites exemplify antibacterial bioactive nanocomposites due to their unique combination of bactericidal and bioactive properties.

**Figure 1:**
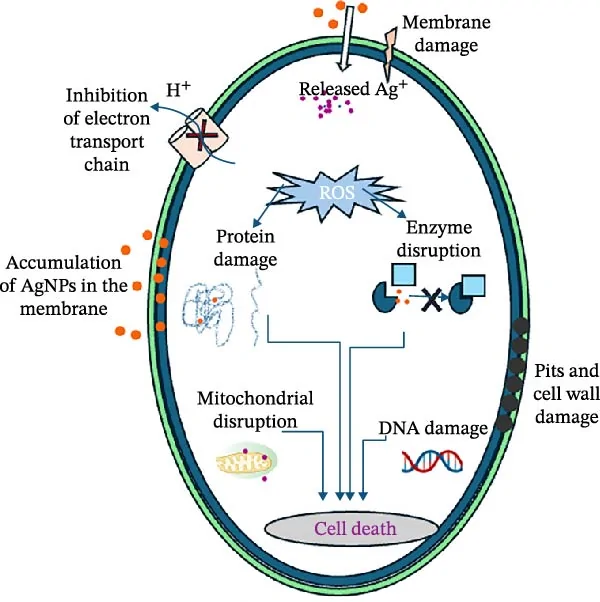
Schematic representation of the antibacterial activity of AgNPs.

Figure 1 illustrates the antibacterial activity of AgNPs, which exert their effects through multiple complementary mechanisms. Ag^+^ ions adhere to or penetrate the bacterial cell wall and cytoplasmic membrane, disrupting these structures. Ribosomes undergo denaturation, which consequently inhibits protein synthesis. Concurrently, the production of adenosine triphosphate (ATP) is impaired due to the deactivation of respiratory enzymes located on the cytoplasmic membrane. This impairment in the electron transport chain results in the formation of ROS, which subsequently inflicts damage and causes disruption to the membrane. Additionally, Ag^+^ ions and ROS interfere with DNA replication by binding to deoxyribonucleic acid, thereby preventing proper cell division. AgNPs may also accumulate in pits within the cell wall, causing membrane denaturation, and they may directly perforate the cytoplasmic membrane, leading to leakage of cellular contents. Collectively, these mechanisms result in rapid and irreversible bacterial cell death. A recent strategy involved combining AgNPs with ciprofloxacin, encapsulating the antibiotic in a AgNP carrier, and dispersing it into a resin composite. Arif et al. [47] reported that a composite containing 0.5% ciprofloxacin–loaded AgNPs (CIP‐AgNPs) demonstrated strong inhibition of *S. mutans* biofilm formation and reduced microleakage at the tooth–restoration interface while maintaining flexural strength and degree of conversion (DC) comparable to those of the control.

**Figure 2:**
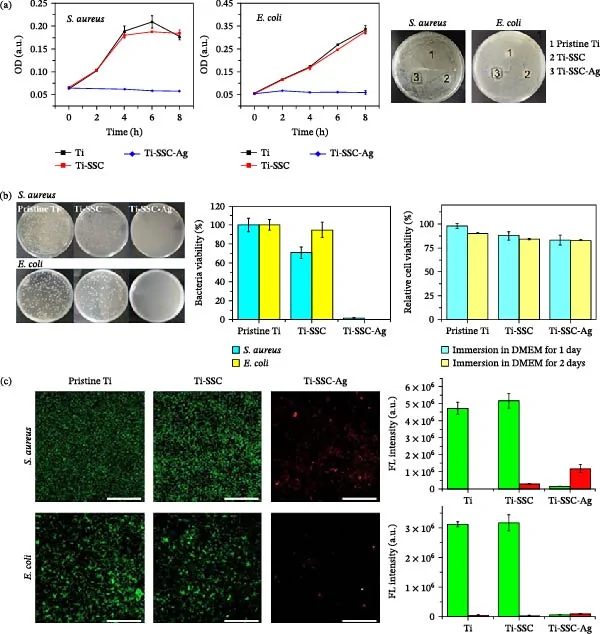
AgNP in antibacterial coating of implants: Growth curves and inhibition zones of (a) *S*. *aureus* and *E. coli* (b) TSB‐agar plate images of the serially diluted bacterial colonies produced by *S. aureus* and *E. coli*; percentage of the colonized viable bacterial cells on the pristine Ti, Ti‐SSC, and Ti‐SSC‐Ag substrates. (c) CLSM micrographs of *S. aureus* and *E. coli* stained with SYTO 9 and PI on the pristine Ti, Ti‐SSC, and Ti‐SSC‐Ag surfaces, as well as live (green) and dead (red) cells on the pristine and modified Ti surfaces (scale bar = 100 μm) [57]. Reprinted with permission.

Figure 2 shows that AgNPs were incorporated as antibacterial coatings for dental implant surfaces. (a) Growth curve analyses and inhibition zone assays illustrate the responses of *Staphylococcus aureus* and *Escherichia coli* exposed to pristine Ti, Ti–silk sericin composite (Ti‐SSC), and Ti‐SSC‐Ag surfaces. (b) TSB agar plate images display bacterial colonies derived from serial dilutions of each strain, along with quantitative evaluations of viable bacterial adhesion on the three substrate types. (c) CLSM images of *S. aureus* and *E. coli* biofilms stained with SYTO 9 (live, green) and PI (dead, red) show the distribution and viability of cells on pristine Ti, Ti‐SSC, and Ti‐SSC‐Ag surfaces. (d) In vitro cytocompatibility measurements of L929 mouse fibroblasts cultured for 1 and 2 days reveal the biocompatibility of each surface modification [58]. Qiang et al. [57] demonstrated that Ti surfaces coated with silk sericin–assisted AgNPs effectively inhibited *S. aureus* and *E. coli* adhesion and early biofilm formation while maintaining excellent cytocompatibility. Subsequent studies using AgNP coatings combined with antimicrobial peptides or silk‐fibroin–based systems further confirmed strong, long‐lasting antibacterial activity, along with enhanced osteogenic responses and improved tissue integration in vivo. Clinical evidence also supports these findings, as AgNP‐coated healing abutments significantly reduced bacterial attachment without causing cytotoxic effects in human gingival fibroblasts [57].

Table 1 summarizes the antimicrobial performance of various AgNP–modified dental materials and their effectiveness against different microorganisms. Although this concept introduces an additional antibiotic component, it demonstrates how NPs can be used as dual‐function antimicrobial and carrier systems [76]. The main drawbacks of Ag‐based nanocomposites are their potential discoloration, especially at higher filler loadings, and dose‐dependent cytotoxicity to fibroblasts and pulp cells [55, 77]. Careful optimization of particle size, loading, and encapsulation within glass or polymer matrices is necessary to balance antibacterial efficacy with aesthetic and biological safety requirements [78]. Although AgNPs consistently demonstrate strong antibacterial activity across a wide range of experimental systems, direct comparisons remain challenging due to substantial variability in NP size, surface modification, concentration, and incorporation methods. Furthermore, most investigations have been limited to short‐term in vitro biofilm models that may not accurately reproduce the complex oral environment.

**Table 1:** Antimicrobial effects of AgNP‐enhanced dental materials.

Material	AgNP concentration	Antimicrobial activity	Ref
Endodontic AgNP irrigants/medicaments	Typically 10‐nm AgNPs with 17% EDTA	AgNP‐based irrigants showed *E. faecalis* killing comparable to 2.5% NaOCl and effective smear layer removal; positively charged AgNPs achieved similar bactericidal action with sometimes lower cytotoxicity than NaOCl	[58]
MTA	1 wt%	The antimicrobial efficacy against *E. faecalis*, *C. albicans*, and *P. aeruginosa* is notably high	[59]
Intracanal irrigant	0.00005	Bactericidal effect against *E. faecalis*	[60]
Tissue conditioner	0.1%, 0.5%, 1.0%, 2.0%, 3.0%	The antimicrobial efficacy was observed against *S. mutans* and *S. aureus* at a concentration of 0.1% and against *C. albicans* at a concentration of 0.5%	[61]
Ti implants	3.16 × 10^−2^ mg Ag/mL	*P. aeruginosa* adhesion reduction	[62]
	0.5 M, 1.0 M, 1.5 M, 2.0 M	*S. aureus* adhesion prevention for up to 30 days	[63]
Composite resin	0.028 wt%, 0.042 wt%, 0.088 wt%, 0.175 wt%	Good inhibitory activity against *S. mutans*, at 0.042 wt%	[64]
Adhesive system	0.05 wt%	Reduction of CFU and lactic acid production for *S. mutans*	[65]
	0.1 wt%	Reduction of CFU for total microorganisms, total streptococci, and *S. mutans*	[66]
Primer and adhesive	0.05 wt%	The compound exhibits significant inhibitory activity against a broad spectrum of microorganisms, including total streptococci and *S. mutans*	[67]
Flowable composite resin	0, 0.02, 0.03, 0.04, 0.05 wt% AgNPs	All AgNP groups showed significant inhibition of *Streptococcus mutans* vs. control; strongest effect at 0.03 wt%	[68]
Acrylic resin	1 µg/mL	Reduction of *C. albicans* adherence	[69]
	0.05 vol%, 0.5 vol%, 5 vol%	Good efficacy against *C. albicans*, at 5 vol%	[70]
Composite resin with CIP‐AgNPs	1 wt% AgNPs, 1 wt% CIP‐AgNPs	1% CIP‐AgNP–modified discs almost completely inhibited *E. faecalis*, *S. mutans*, and saliva microcosm; stronger than 1% AgNPs; compressive strength improved	[47]
Self‐etch dentin adhesive (Clearfil SE Bond, Kuraray Noritake Dental Inc., Tokyo, Japan)	0.05 wt% AgNPs or 0.05 wt% Ag@nGO	Significant reduction in viable *S. mutans*, lactic acid production, inhibition zones, and CFUs vs. control	[71]
PMMA denture base resin	0.1, 1, 10 wt% AgNPs	AgNP‐ and especially 10% Nys‐coated AgNP–PMMA showed strong antifungal activity vs. *Candida albicans* and antibacterial effect vs. *S. mutans* with no relevant cytotoxicity	[72]
Denture glaze (Palaseal, Kulzer GmbH, Hanau, Germany)	AgNPs (UV, Turkevich)	AgNP‐glaze groups showed marked reductions in viable *C. albicans* cells and metabolic activity up to 30 days; anti‐Candida effect comparable to glaze+nystatin, nontoxic to fibroblasts	[73]
Silver‐coated Ti implant abutment discs	Silver coating releasing ~0.34 mg/L Ag after 24 h	Dissolved silver significantly reduced planktonic *S. mutans* growth and lactate production vs. uncoated controls; no significant antibiofilm effect on established biofilms	[74]
Ti implants with PEO Ag/Zn coating	Ag and Zn	Mixed anaerobic biofilm on Ag/Zn‐loaded implants showed ~2‐log (99%) CFU reduction and ~5‐log (99.999%) DNA reduction vs. unloaded Ti controls	[75]

### 4.2. Other Metallic NPs

#### 4.2.1. Copper NPs (Cu NPs)

**Figure 3:**
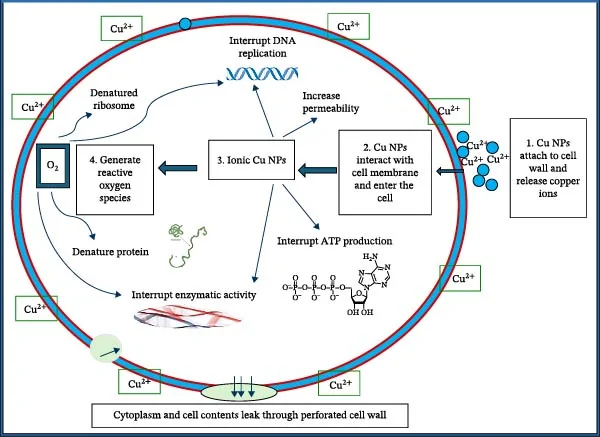
Antibacterial mechanism of Cu NPs.

Cu NPs (Cu or CuO) have also been investigated as antibacterial fillers for dental materials [79]. Their antimicrobial effect is attributed to Cu^2+^ release, ROS generation, and membrane damage, with low cost being an advantage over Ag. There are fewer studies specifically on Cu‐containing restorative composites than on Ag or zinc; however, in vitro studies have shown that CuO NP incorporation into experimental composites or sealants can significantly reduce *S. mutans* growth and biofilm formation, although color stability and cytotoxicity remain concerns [34, 35, 80]. Figure 3 represents the antibacterial activity of Cu NPs.

Recent studies have demonstrated that incorporating Cu‐based NPs into restorative composites, adhesives, polymethyl methacrylate (PMMA) resins, tissue conditioners, and glass‐ionomer–based materials can markedly reduce *S. mutans* growth and biofilm formation, while some systems also show activity against *Enterococcus faecalis*, *Pseudomonas aeruginosa*, and *Candida albicans* [81–83]. Table 2 presents the properties and applications of dental materials incorporating Cu NPs.

**Table 2:** Properties and applications of dental materials incorporating Cu NPs.

Materials	Properties	Applications	Ref
Cu‐doped mesoporous BG nanosphere acrylic resin	Facilitate Cu‐ion release; antimicrobial activity	Denture acrylic base	[83]
Cu NPs	Anti‐inflammatory activity	Periodontal therapy	[84, 85]
Cu NPs in glass ionomer cement	Offer antimicrobial properties	Glass ionomer restoration	[86]
Polyacrylic acid–Cu iodide NPs with adhesive resin	Offer antimicrobial properties	Dental adhesive	[87]
Ti–hydroxyapatite–Cu alloy	Offer antimicrobial properties	Dental implant	[88]
Cu NPs with adhesive resin	Offer antimicrobial properties	Dental adhesive	[89]
CuO–nanoparticle–modified tissue conditioner	Antibacterial and antifungal	Tissue conditioner	[82]
Nano Cu–nonstoichiometric dicalcium silicate	Antimicrobial and tissue regenerative	Regenerative material	[90]
Cu NP denture adhesive	Antimicrobial	Denture adhesive	[91]
Cu‐doped mesoporous bioactive glass NP composite	Ion release; antibacterial; remineralizing	Restorative composite resin	[86]
Functionalized Cu phosphate NPs	Enhance vascularization and bone regeneration	Regenerative cement	[92]
Graphene oxide Cu nanocomposite	Enhance bone regeneration	Regenerative material	[93]
Cu‐nanoparticle–incorporated acrylic resin	Offer antimicrobial properties	Denture soft liner	[94]

#### 4.2.2. Gold NPs (AuNPs)

AuNPs have primarily been explored for coatings and scaffolds rather than for bulk composites due to cost considerations [95]. Nevertheless, their chemical stability, ease of functionalization, and plasmonic properties have led to experimental AuNP–modified resins with promising antibacterial and cell‐compatible behaviors, albeit still at the proof‐of‐concept stage [96]. AuNP incorporated into various dental materials has demonstrated strong antibacterial effects, effectively reducing the growth and adhesion of key oral pathogens like *S. aureus*, *E. coli*, *Porphyromonas gingivalis*, and *C. albicans* across multiple modification techniques, as shown in Table 3.

**Table 3:** Antibacterial effects of AuNPs in dentistry.

Material with AuNP and technique	Antibacterial property demonstrated	Ref
Ti–magnetron sputtering (20–26 nm, spherical AuNPs)	Restricted growth of *E. coli* and *S. aureus* without cytotoxicity	[97]
Ti–UV‐light decomposition of HAuCl_4_	Enhanced antibacterial properties against multispecies biofilm through photocatalytic production of ROS	[98]
Ti–UV‐light decomposition of HAuCl_4_ (20 nm AuNPs on TNTs)	Significantly increased antibacterial activity against *P. gingivalis* through ROS under ultrasound	[99]
Ti–magnetron sputtering with tetracycline/polycaprolactone (TC/PCL)	Antibacterial activity with NIR‐triggered tetracycline release	[100]
PMMA–AuNP suspension in monomer (57–82 nm, spherical)	Strong antibiofilm effect against *C. albicans*, *S. mitis*, *S. aureus*, *E. coli*	[101]
PMMA–AuNP suspension in monomer (11 nm, spherical)	Reduced adhesion of *C. albicans*	[102]
Polyurethane resins (aligners)—AuDAPT coating (<4 nm)	Antibacterial effect against *P. gingivalis*; inhibited biofilm formation	[103]
Stainless steel–BOA–AuNP nanocomposite (3.5–5.5 nm)	Decreased *S. mutans* colonization on orthodontic appliances	[104]

## 5. Metal Oxide NPs

### 5.1. Zinc Oxide NPs (ZnO NPs)

**Figure 4:**
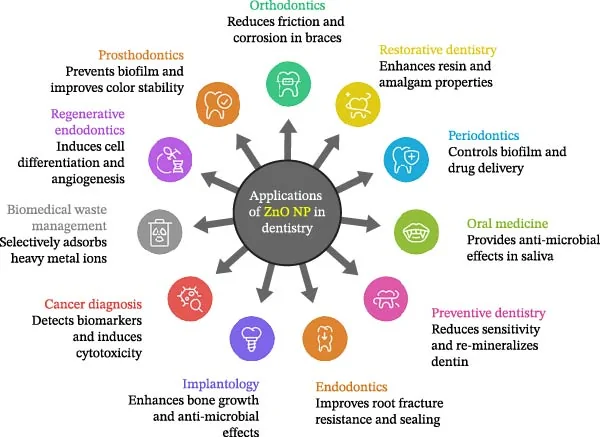
Schematic representation illustrating the application of ZnO NP in dentistry.

ZnO NPs are among the most extensively studied antibacterial fillers for dental applications. ZnO NPs exert antimicrobial activity by releasing Zn^2+^ ions and generating ROS and by direct interaction of NPs with bacterial membranes [105]. A systematic review by Garcia et al. [106] evaluated the effect of ZnO NP incorporation on direct composite resins and concluded that the majority of in vitro studies reported statistically significant reductions in bacterial viability or biofilm formation against *S. mutans* or *Lactobacillus* strains when ZnO NPs were added at concentrations between 1 and 10 wt%. The review also noted that mechanical outcomes were variable: Low‐to‐moderate loadings sometimes preserved or slightly improved flexural properties, whereas higher loadings or poor dispersion tended to decrease strength and toughness. Figure 4 illustrates the applications of ZnO NP in dentistry.

Applications of ZnO NPs in dentistry include their use in restorative materials, adhesives, cements, endodontic medicaments, and denture bases to provide strong antibacterial and antibiofilm effects, as shown in Figure 4. Al‐Mosawi et al. [46] incorporated 1–5 wt% ZnO NPs into a microhybrid composite and demonstrated significant inhibition of *S. mutans* and *Lactobacillus acidophilus* in direct contact and agar diffusion tests, with the strongest effect at 5 wt%. The flexural strength only decreased at the highest loading, suggesting a relatively wide therapeutic window.

Table 4 summarizes recent studies demonstrating the antibacterial performance of ZnO NP–modified dental materials across various clinical applications. Wang et al. [105] designed cellulose nanocrystal/ZnO nanohybrids, in which Zn^2+^ was precipitated onto cellulose nanocrystals (CNCs) before incorporation into a Bis‐GMA/TEGDMA resin. This configuration improved the NP dispersion and interfacial bonding. Composites with 3 wt% CNC/ZnO showed significantly enhanced flexural strength and modulus compared with the unmodified resin, as well as a pronounced reduction in *S. mutans* CFUs and lactic acid production over 7 days [105]. Recently, tetrapod‐shaped ZnO (t‐ZnO) whiskers have been incorporated into dual‐cure resin composites. In an experimental system containing up to 20 wt% tZnO, composites with 5–10 wt% tZnO displayed the best balance among improved flexural strength, reduced water sorption, and potent antibacterial activity against *S. mutans*, whereas higher loadings led to excessive brittleness [2, 112]. The findings underscore the impact of particle morphology on both mechanical and biological performance. In addition to simple ZnO, zinc‐doped BG NPs have also been incorporated into etch‐and‐rinse adhesives. Choi et al. [37] synthesized mesoporous BG NPs (MBNs) doped with zinc (MBN‐Zn) and showed that adhesives containing 5 wt% MBN‐Zn significantly inhibited MMP‐mediated collagen degradation, enhanced the micro tensile bond strength after thermocycling, and exhibited antibacterial activity against *S. mutans*. This dual action at the resin–dentin interface is an example of how zinc can contribute simultaneously to antibacterial and host‐modulating effects.

**Table 4:** Antibacterial effects of ZnO NP in dentistry.

Material (ZnO NP modified)	Antibacterial effect	Ref
Self‐curing acrylic resin with ZnO NPs	Inhibited *Streptococcus mutans* biofilm formation	[107]
Toothpaste containing ZnO NPs	Increased inhibition of *S. mutans* in a concentration‐dependent manner	[108]
Soft denture liner with ZnO NPs	Significant reduction of *Candida albicans* growth	[109]
PMMA denture base with ZnO/ZnO‐Ag NPs	Antifungal activity against *C. albicans*	[110]
Resin composites with rod‐like ZnO NPs	Strong antibacterial activity against *S. mutans*	[111]
Dual‐cure resin composites with t‐ZnO whiskers	Enhanced antibacterial effect and good cytocompatibility	[112]
Ti implants with ZnO nanorod drug‐loaded nanocoating	Antibacterial via Zn^2+^ release and ROS production	[113]
Orthodontic adhesive with ZnO NPs	Reduced biofilm formation while maintaining bond strength	[114]
Ti implants functionalized with Ca‐doped ZnO NPs	Antibacterial surface with improved osteoblast response	[115]
Review summarizing ZnO NP antibacterial dental applications	Confirms activity across restorative, endodontic, prosthodontic, implant, and orthodontic materials	[116]

Despite the extensive literature supporting the antibacterial efficacy of ZnO NPs, considerable heterogeneity exists among studies regarding NP morphology, loading concentration, bacterial models, and testing methodologies. This variability complicates the establishment of optimal formulations and limits direct comparison between investigations. Moreover, improvements in antibacterial performance are frequently accompanied by trade‐offs in mechanical properties at higher NP concentrations. Future studies should focus on standardized testing protocols and clinically relevant biofilm models to better define the therapeutic window for ZnO‐based restorative materials.

### 5.2. Titanium Dioxide NPs (TiO_2_ NPs)

TiO_2_ NPs (TiO_2_‐NPs) are chemically stable and white, making them aesthetically appealing [117]. They possess intrinsic antibacterial properties primarily through the generation of cytotoxic oxygen radicals [118, 119]. Their antimicrobial activity is mainly photocatalytic when excited by ultraviolet (UV) or visible light. Upon photoexcitation, electrons in the valence band of TiO_2_ are promoted to the conduction band, generating electron–hole pairs. These charge carriers react with oxygen and water molecules to form ROS such as hydroxyl radicals (•OH), superoxide anions (O_2_•^−^), and hydrogen peroxide, which exert strong oxidative stress on bacterial cells and lead to membrane disruption, protein oxidation, and DNA damage [120]. TiO_2_ generates ROS such as hydroxyl radicals and superoxide, which damage bacterial membranes, proteins, and DNA [121]. To shift the absorption spectrum from the UV to the visible‐light region, TiO_2_ can be doped with nitrogen to reduce its band‐gap energy [122, 123]. However, the relatively wide band gap of TiO_2_ (~3.2 eV) limits its activation primarily to UV light. To overcome this limitation, nitrogen doping has been explored as an effective strategy to narrow the band gap and shift the absorption spectrum toward the visible‐light region, allowing photocatalytic activation under clinically relevant lighting conditions [124]. Rodrigues et al. developed nitrogen‐doped TiO_2_ (N‐TiO_2_) NPs and incorporated them into experimental dental adhesives. They demonstrated that N‐TiO_2_ thin films exhibited increased hydrophilicity and sustained ROS generation under visible light, which significantly reduced the viability of *S. mutans* biofilms compared to unmodified adhesives [125]. Subsequent work demonstrated that incorporating 10–20 vol% N‐TiO_2_ into a commercial adhesive enhanced the DC and biaxial flexural strength while providing long‐term antibacterial functionality without adversely affecting sorption and solubility [122, 126]. Fauzi et al. [123] examined N‐TiO_2_ as an aesthetic antimicrobial filler in a resin matrix and reported that resin discs containing 5–10 wt% N‐TiO_2_ preserved translucency and color while displaying robust antibacterial effects against *S. mutans* under visible light. These findings suggest that N‐TiO_2_ may be particularly suitable for anterior composites and adhesives where color stability is critical. These studies collectively suggest that N‐TiO_2_ can impart visible‐light–activated antibacterial properties to dental materials while preserving their mechanical integrity and aesthetic qualities. This supports its potential application as a multifunctional additive in restorative and adhesive systems. Although photocatalytic TiO_2_ systems demonstrate promising antibacterial activity under controlled laboratory irradiation, their clinical effectiveness may be limited by the low and intermittent availability of activating light within the intraoral environment.

**Figure 5:**
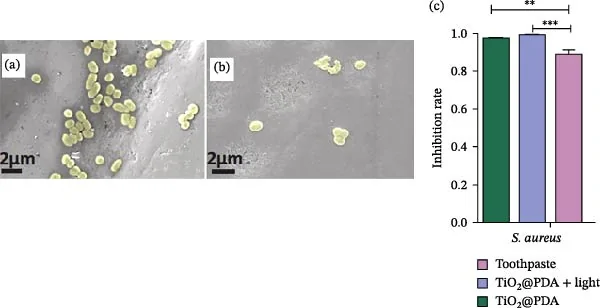
Comparative antibacterial efficacy of (a) commercially available toothpaste, (b) TiO_2_/PDA composite in the absence of light irradiation, and (c) inhibition rates of various treatments against *Staphylococcus aureus* [127]. Data are presented as mean ± standard deviation:  ^∗∗^ indicates *p* < 0.01 and  ^∗∗∗^ indicates *p* < 0.001. Reprinted with permission.

Figure 5 represents the comparative antibacterial efficacy of a toothpaste with TiO_2_ and its antibacterial activity. Beyond adhesives, TiO_2_ NPs have been investigated in orthodontic resins and acrylics. A BMC Oral Health study found that incorporating TiO_2_‐NPs into orthodontic acrylic appliances resulted in statistically significant reductions in biofilm accumulation and *S. mutans* counts, although mechanical behavior depended strongly on NP concentration and surface treatment [128]. Recent research has explored the codoping of TiO_2_ with zinc and nitrogen to further enhance antibacterial performance and visible‐light responsiveness, potentially yielding dual‐function photocatalytic and Zn^2+^‐releasing fillers [122, 129]. Giti et al. [130] demonstrated that integrating TiO_2_ NPs into PMMA denture base resin significantly enhanced its antibacterial properties, with a 7.5 wt% TiO_2_ concentration notably inhibiting the growth of *C. albicans*.

In their study, Zhang et al. [127] demonstrated that the TiO_2_/polydopamine (PDA) composite exhibited significant antibacterial properties against *S. aureus*. This effect is largely attributed to the presence of TiO_2_, which has a strong ability to generate ROS even in the absence of external light activation [127]. The application of a PDA coating significantly improved the dispersion and stability of TiO_2_ NPs and promoted efficient electron transfer. This enhancement led to increased ROS production, thereby augmenting the bactericidal properties. In a study by Florez et al. [125], N‐TiO_2_ NPs were integrated into a commercial adhesive, resulting in a notable decrease in *S. mutans* cariogenic biofilm formation under dark conditions, exceeding the efficacy of the unmodified adhesive resin.

**Figure 6:**
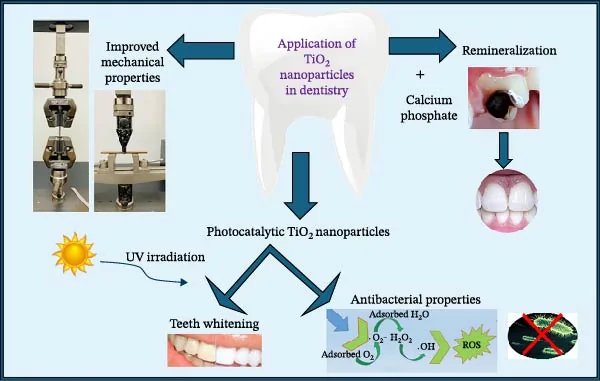
Various applications of TiO_2_ NPs in dentistry.

Figure 6 depicts the diverse applications of TiO_2_ NPs in dentistry. In addition to their intrinsic antibacterial properties, TiO_2_ NPs exhibit strong photocatalytic antimicrobial effects under light irradiation. When subjected to blue or UV light, especially at wavelengths under 385 nm, there is an increase in the generation of ROS due to the photoexcitation of TiO_2_ in the presence of oxygen and moisture [131, 132]. The species generated, such as hydrogen peroxide, superoxide anions, and hydroxyl radicals, exhibit significant oxidative potential, allowing them to effectively and rapidly neutralize microorganisms [133]. Kotta et al. [134] integrated 1 wt% TiO_2_ NPs into a standard resin, resulting in a marked decrease in the formation of *S. mutans* biofilms. Sodagar et al. [135] demonstrated that the addition of 1 wt%, 5 wt%, and 10 wt% TiO_2_ NPs to Transbond XT adhesive resulted in a dose‐dependent decline in *S. mutans* colonies over a 3‐day period. The incorporation of 10 wt% TiO_2_ NPs into GIC in orthodontic applications has been demonstrated to significantly enhance its antibacterial efficacy against *S. mutans* [136]. Behnaz et al. [137] demonstrated that the incorporation of 2 wt% TiO_2_ NPs into Transbond X significantly reduced the incidence of white spot lesions (WSLs) following a 1‐month clinical evaluation. Ghasemi et al. [138] demonstrated that incorporating TiO_2_ NPs onto stainless steel brackets led to a substantial reduction in bacterial colony counts compared with uncoated brackets. Utilizing atomic layer deposition (ALD) to apply TiO_2_ coatings on PMMA has been demonstrated to markedly reduce the attachment of *C. albicans* in comparison to PMMA that has not been modified [138]. Sodagar et al. [135] reported that integrating 10 wt% TiO_2_ NPs into an orthodontic resin composite resulted in a marked decrease in *S. mutans* counts compared with the unmodified resin. Orthodontic adhesives and primers, which bind brackets to tooth enamel, are also known contributors to WSL formation due to acidogenic biofilm accumulation at bracket margins [139]. Although TiO_2_‐based materials show promising antibacterial effects in laboratory settings, certain limitations must be considered for their clinical application. The effectiveness of the photocatalytic activity of TiO_2_ is also related to the wavelength and intensity of light that can be encountered in the oral cavity, which may not always be fulfilled. Light activation is frequently restricted and intermittent and may be affected by the location of the restoration, the amount of coverage provided by the restoration, the presence of saliva, the surrounding soft tissues, and the patient’s characteristics in clinical situations. Therefore, the in vivo antibacterial performance observed under continuous laboratory irradiation might be overstated. Several strategies have been employed to enhance the material’s visible‐light responsiveness, including nitrogen doping and codoping, but further study is needed to determine whether effective photocatalytic activation can be maintained under clinically relevant oral conditions to provide a clinically relevant antibacterial effect.

### 5.3. MgO‐NPs

MgO‐NPs exhibit high surface basicity, which can raise the local pH, disrupt bacterial cell walls, and interfere with cellular respiration. Their degradation products are biocompatible, which makes MgO an attractive candidate for biomedical applications [128, 140, 141].

**Figure 7:**
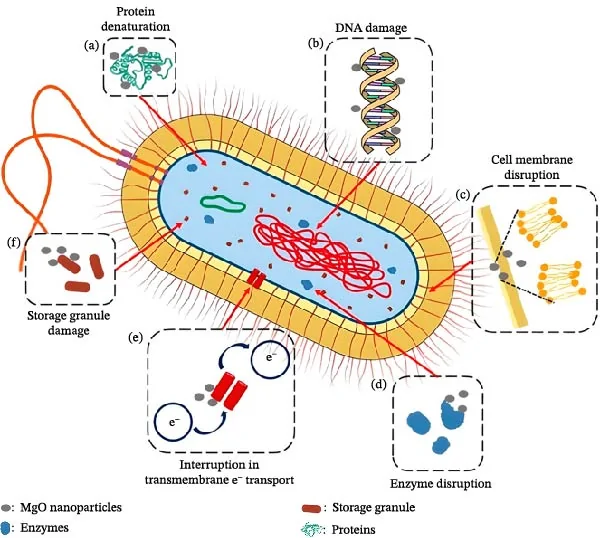
Potential antibacterial actions of MgO‐NPs. After interacting with and penetrating the bacterial envelope, MgO NPs may induce protein denaturation (a), DNA damage (b), and cell‐membrane disruption (c). They can also impair enzyme function (d), interrupt transmembrane electron transport (e), and damage intracellular storage granules (f), collectively leading to bacterial‐cell death [142] (Under CC License).

Figure 7 shows the schematic representation of the proposed antibacterial mechanism of MgO‐NPs. The disruption of the bacterial membrane allows MgO‐NPs to penetrate the cell (c). Once inside, these NPs can either promote the formation of ROS or cause direct harm to intracellular components, such as DNA and vital enzymes (b,d). These disturbances lead to protein denaturation and mitochondrial dysfunction (a,f). Additionally, the NPs may disrupt cellular memory processes and obstruct trans‐tolerant electron transport pathways (e). These combined effects compromise cellular integrity, leading to organelle leakage and ultimately resulting in the death of the bacterial cell [142]. Sharifian et al. [140] conducted a review detailing the antibacterial and physical properties of MgO‐NPs when applied to various dental substrates, highlighting their superior biocompatibility compared to numerous other oxides. In an investigation, MgO‐NPs were incorporated into an experimental Bis‐GMA/TEGDMA composite at varying concentrations ranging from 1 to 15 wt%. The study demonstrated a dose‐dependent enhancement in antibacterial efficacy against *S. mutans* and *E. faecalis*. Notably, composites containing 2–5 wt% MgO achieved an optimal balance between flexural strength, hardness, and antibacterial effectiveness [143, 144]. MgO‐NPs have been added to denture base resins and 3D‐printed resins [24], where they significantly reduce colonization by *C. albicans* and mixed biofilms while maintaining or modestly improving the flexural modulus and hardness at low concentrations [145].

Table 5 summarizes the recent antibacterial applications of MgO‐NPs in dentistry, highlighting their effectiveness against key oral pathogens across various dental materials and treatment modalities.

**Table 5:** Antibacterial effects of MgO‐NPs in dentistry.

Material with MgO‐NP	Antibacterial activity	Method/application	Ref
NPs (powder/colloidal form)	Strong antibacterial activity against *Streptococcus mutans*, *Staphylococcus aureus*, *Enterococcus faecalis*, *Escherichia coli*	MIC testing, CFU reduction, biofilm inhibition assays	[146]
Flexible denture base (coated or impregnated)	Significant inhibition of *Streptococcus mutans* biofilm	Coated vs. impregnated denture material tested in vitro	[147]
Green‐synthesized NPs	Against *S. mutans*, *S. aureus*, *E. faecalis*	Agar diffusion, CFU reduction, ROS‐based antibacterial mechanism	[148]
Glass‐ionomer cement (modified)	Reduced *S. mutans* growth and biofilm formation	Modified GIC tested via agar diffusion and antibiofilm assays	[149]
Ti implant surface (coated)	Strong antibacterial activity against *Porphyromonas gingivalis*	Electrophoretic deposition coating; antibacterial testing and SEM	[150]

## 6. Polymeric and Organic NPs

### 6.1. QA‐PEI NPs

The QA‐PEI NPs are derivatives of cross‐linked polyethylenimine (PEI), modified with quaternary ammonium groups. Their surfaces carry a high density of positive charges, which interact with the negatively charged bacterial membranes, leading to membrane disruption and leakage of cytoplasmic contents. Because QA‐PEI is covalently cross‐linked, it shows negligible leaching, and its antibacterial effect is predominantly contact‐active [5, 7, 8]. Beyth et al. [5] were the first to embed QA‐PEI NPs (1 wt%) into commercial flowable, hybrid, and bonding composites and to evaluate their antibacterial effect against *S. mutans* using a direct contact test. They reported complete inhibition of *S. mutans* growth for at least 3 months of aging in water, without significant reduction in flexural strength, modulus, or DC compared with unmodified materials [5]. Subsequent studies analyzed how the alkyl chain length of the quaternary substituents influences the activity; octyl‐alkylated QA‐PEI showed stronger antibacterial action than shorter‐chain derivatives, indicating that both charge density and hydrophobicity modulate membrane interactions. Chladek et al. [7] recently prepared experimental dimethacrylate–based composites containing 0.5–3 wt% QA‐PEI NPs and assessed bacterial adherence, cytotoxicity, and mechanical properties. Composites with 1 wt% QA‐PEI significantly reduced *S. mutans* adherence and lactic acid production while demonstrating acceptable flexural strength, water sorption, and cytocompatibility with human fibroblasts. Higher loadings improved antibacterial action but began to compromise mechanical performance and increased cytotoxicity in some assays [7]. A review of QA‐PEI NPs as biocidal additives concluded that these fillers are among the most promising nonleaching antibacterial nanofillers for dental composites and cements because of their strong contact killing, compatibility with dimethacrylate matrices, and relatively low toxicity in vitro [8]. Table 6 summarizes various notable studies of QA‐PEI NPs used in dentistry. QA‐PEI is engineered into crosslinked NPs with dimensions ranging from several tens to a few 100 nanometers. These NPs are incorporated at low concentrations, typically around 1 wt%, into dimethacrylate‐based dental composites and other restorative materials. This integration provides potent and durable contact‐killing properties without significant leaching and with minimal negative effects on the DC, mechanical properties, or biocompatibility [8]. QA‐PEI NPs have gained attention as effective nonleaching antibacterial agents for a range of dental materials, especially restorative composites. They offer the potential to extend the lifespan of restorations and decrease the occurrence of secondary caries. Despite promising antibacterial findings, current evidence supporting QA‐PEI‐ and chitosan‐based systems remains predominantly preclinical, with limited ex vivo and minimal clinical validation available to date.

**Table 6:** Antibacterial effects of QA‐PEI NPs in dentistry.

Dental material/system	QA‐PEI/QPEI NP (formulation and loading)	Antibacterial action	Ref
Commercial dimethacrylate composites: Filtek Z250 (hybrid), Filtek Flow (flowable), and bonding resin	Cross‐linked QA‐PEI NPs, 1 wt% in resin	0.5–2 wt% QA‐PEI NPs provide efficient, long‐lasting antibacterial effects (mostly against *S. mutans*, but also *S. aureus*, *E. faecalis*, *P. aeruginosa*, etc.) without elution	[151]
Experimental Bis‐GMA/TEGDMA‐based composite resins	QA‐PEI NPs synthesized by crosslinking + N‐alkylation (octyl) + N‐methylation; typically 1–2 wt% in composite	QA‐PEI NPs completely inhibited *S. mutans* growth in vitro at low loadings; antibacterial activity strongly depended on degree of N‐alkylation and quaternization	[152]
Provisional PMMA‐based cements	Cross‐linked PEI NPs QPEI/QA‐PEI at 0.5, 1, and 2 wt%	Strong antibacterial effect against *S. mutans* and *E. faecalis* for ≥14 days; significant inhibition from ≥1 wt% loading	[152]
Experimental resin composites	Insoluble cross‐linked QA‐PEI NPs blended into resin matrix (1–2 wt%)	Broad surface antimicrobial spectrum against oral bacteria (*S. mutans*, salivary flora, *Staphylococcus aureus*, *Staphylococcus epidermidis*, *Enterococcus faecalis*, *Pseudomonas aeruginosa*, and *Escherichia coli*); durable contact‐killing with no detectable leaching	[153]
Glass ionomer cements used for crowns	QPEI NPs (quaternary ammonium PEI) incorporated into GI cements	Long‐lasting antibacterial action against *S. mutans* and *Lactobacillus casei*; substantial reduction in bacterial counts vs. control cements	[154]
Resin‐based dental materials (experimental composites and sealers)	Multiple QPEI NP formulations differing in crosslinking, alkyl chain, and quaternization degree; ~2 wt% typical	All QPEI variants gave prolonged and complete inhibition of bacterial growth when incorporated in resin materials; potency depended strongly on synthesis route (alkyl chain and degree of quaternization)	[155]
Neobond orthodontic bracket cement (Dentsply, York, PA, USA) on extracted teeth	QPEI NPs in orthodontic cement	Ex vivo bracket model: cement with QPEI significantly reduced *S. mutans* and *L. casei* CFUs and OD around brackets vs. control cement	[156]
Orthodontic adhesive system for bracket bonding	QPEI NPs at 1 and 1.5 wt%	Long‐lasting antibacterial activity against *S. mutans* after 14‐day aging; strong reduction in viable bacteria vs. unmodified adhesive	[157]
Experimental Bis‐GMA/UDMA/TEGDMA composites with glass fillers	Cross‐linked QA‐PEI NPs 0.5–3 wt%	All QA‐PEI–modified composites showed complete reduction of adhered *S. mutans* and satisfactory biocompatibility (MTT, 48 h, and 10‐day extracts); contact‐killing, nonleaching behavior	[7]

### 6.2. Chitosan‐Based NPs

Chitosan is a cationic polysaccharide derived from chitin through deacetylation, possessing inherent antibacterial, bioadhesive, and hemostatic properties [158]. Its primary amine groups become protonated under physiological conditions, enabling electrostatic interactions with microbial membranes and disrupting membrane integrity. Chitosan is a versatile platform for antibacterial bioactive composites, whether processed into NPshen combined with other fillers [159]. A recent review of chitosan‐modified dental resin composites identified more than 20 in vitro studies involving micro‐ or NPs of chitosan as additives for restorative composites, adhesives, or sealants [160]. At low concentrations (typically ≤1 wt%), chitosan‐based particles tended to reduce *S. mutans* adherence and biofilm formation while preserving flexural strength and hardness; at higher concentrations, however, mechanical properties deteriorated, probably due to poor interfacial bonding and increased water sorption [161].

**Figure 8:**
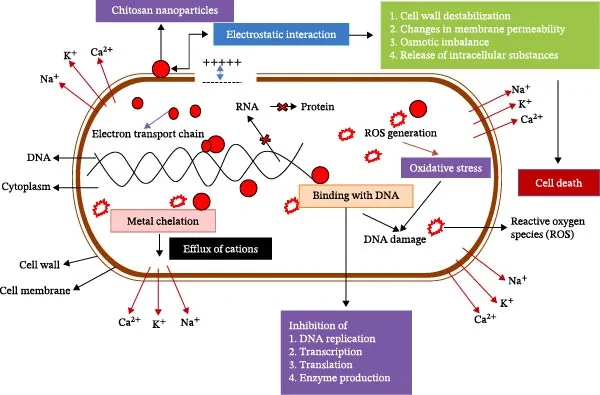
Schematic representation of the antibacterial properties of chitosan NP [170] (Under CC License).

At low levels of incorporation in dental adhesives or restorative materials, chitosan can provide antibacterial properties without significantly altering the physicochemical characteristics of the host substance [162, 163]. The concentration of chitosan plays a crucial role in the overall functionality of the adhesive system. Several studies have indicated that increased chitosan loading can adversely affect adhesive properties, particularly dentin bond strength [164, 165]. Various factors have been identified as contributing to this reduction in adhesion, including the disruption of resin polymerization, increased viscosity of the adhesive system, and degradation of the resin matrix network, which may impede monomer infiltration into demineralized dentin and hinder the formation of hybrid layers [166–168]. The excessive use of chitosan particles may also impact the conversion rate and the mechanical integrity of the adhesive interface [160, 169]. Therefore, while the addition of chitosan offers significant potential to enhance antimicrobial properties, the level of incorporation requires careful optimization to ensure that the compound’s antibacterial efficacy is balanced with adequate bond strength and adhesive stability. However, at higher concentrations, the mechanical and adhesive properties of resin‐based materials may deteriorate. This effect has been attributed to poor interfacial bonding between chitosan particles and the resin matrix, increased water sorption, and possible interference with resin polymerization and monomer infiltration into dentin, which can compromise hybrid layer formation and bond strength. Lucia et al. prepared composite resins containing 0.5 (w/v)% chitosan/dicalcium phosphate anhydrous (DCPA) particles cross‐linked for different durations. Formulations with 0.5 wt% chitosan/DCPA cross‐linked for 16 h showed a significant reduction in biofilm formation without any adverse effect on flexural strength, modulus, or DC compared with the control [161]. Figure 8 schematically represents the antibacterial properties of chitosan NPs.

**Figure 9:**
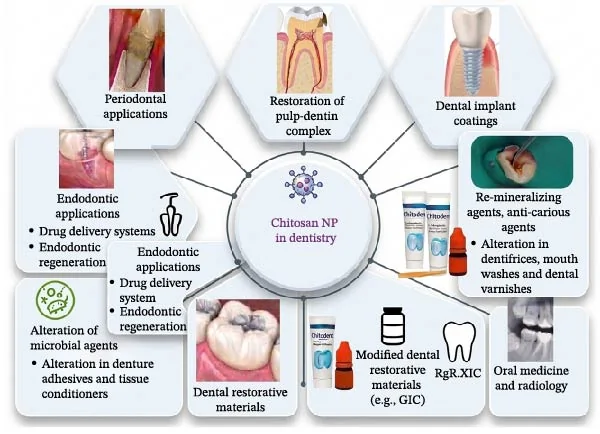
The application of chitosan NP and its derivatives in dentistry.

Figure 9 schematically represents the applications of chitosan‐based NPs in dentistry.

Chitosan has also been combined with ZnO or fluoride to form hybrid antibacterial remineralizing fillers [171]. Mosharrafian et al. fabricated chitosan‐encapsulated ZnO NPs and incorporated them into an adhesive resin. Adhesives containing ZnO–chitosan nanocomposites exhibited stronger reductions in bacterial colony counts and biofilm thickness than adhesives with ZnO alone, likely due to synergistic effects between cationic chitosan and Zn^2+^ release [140, 172]. Lai et al. [173] developed a composite pit‐and‐fissure sealant containing chitosan–fluoride microparticles. The experimental sealant displayed sustained fluoride release, significantly lower *S. mutans* counts, and enhanced microhardness of adjacent enamel blocks after pH cycling compared with a commercial control sealant [173]. This work demonstrates the potential of chitosan to serve as both an antibacterial agent and a carrier for remineralizing ions in preventive restorations. A study that incorporated chitosan and TiO_2_ NPs into resin composites further confirmed the synergistic antibacterial effect of combining organic and inorganic cationic/photocatalytic components while highlighting the importance of optimizing the total filler load to avoid excessive increases in water sorption and decreases in flexural strength [174]. QA‐PEI‐ and chitosan‐based systems may influence long‐term hydrolytic stability, water sorption, matrix degradation, and wear resistance, highlighting the need for further long‐term durability evaluation under clinically relevant conditions.

## 7. Ion‐Releasing Bioactive NPs (BG and S‐PRG Fillers)

BGs and related ion‐releasing fillers bridge the gap between the antibacterial and remineralizing strategies. When exposed to aqueous media, they release Ca^2+^, PO_4_^3−^, F^−^, Sr^2+^, and other ions; increase the pH; and induce the formation of a hydroxycarbonate apatite layer on their surfaces [11, 35, 36, 123]. Ag‐BG has been described above in the context of metallic NPs; however, its bioactive nature is equally crucial. In the Ag‐BG systems developed by Chatzistavrou et al. [175], the incorporation of Ag did not significantly impair the glass’s capacity to form apatite in SBF. Furthermore, the resulting composites exhibited both antibacterial and remineralizing properties [175]. S‐PRG ionomer fillers are produced through an acid–base reaction between fluoroaluminosilicate glass and polyacrylic acid, followed by pulverization. These fillers release multiple ions, including F^−^, Sr^2+^, Na^+^, BO_3_^3−^, and SiO_3_^2−^, which confer anticariogenic and acid‐buffering effects [35, 123]. Experimental composites incorporating S‐PRG fillers at various concentrations have exhibited a reduction in bacterial proliferation on specimen surfaces. Furthermore, there is a correlation between ion release and antibacterial efficacy [35].

**Figure 10:**
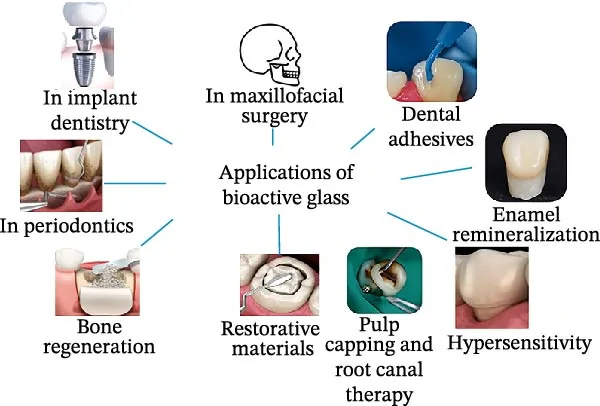
Schematic illustration of the application of BG NPs in dentistry.

Figure 10 illustrates the utilization of BG [176] in the field of dentistry. Recently, composites containing S‐PRG ionomer, specifically Beautifil II, Beautifil LS, and Beautifil Bulk (Shofu Inc.Kyoto, Japan), have been evaluated using a 39‐species subgingival biofilm model associated with periodontitis. Turssi et al. observed that S‐PRG composites significantly altered the microbial profile, reducing the counts of certain periodontopathogens compared to a nonbioactive control composite [36, 177]. Complementary work using S‐PRG–containing tooth‐coating materials demonstrated suppression of periodontopathogenic biofilms and reduced enamel demineralization in an in vitro pH–cycling system [48, 177]. Zinc‐containing phosphate‐based glasses and nanoglasses represent another class of ion‐releasing filler [178]. Deng et al. [38] developed a rapidly dissolving phosphate glass specifically engineered for the release of Zn^2+^ ions and integrated it into a cavity‐liner resin. The material exhibited high Zn^2+^ release, strong inhibition of *S. mutans*, and maintained compressive strength suitable for liner application [38]. Collectively, these studies suggest that BG and S‐PRG NPs can endow composites with indirect antibacterial activity by modulating the ionic environment and pH, in addition to directly influencing biofilm composition, while simultaneously supporting the remineralization of adjacent enamel and dentin.

Table 7 summarizes the approximate NP loading ranges associated with favorable antibacterial performance and highlights the corresponding mechanical and physicochemical trade‐offs reported in the literature. Ion‐releasing bioactive NPs such as BG and S‐PRG fillers often exhibit predominantly indirect antibacterial effects through pH buffering, acid neutralization, and ionic modulation of the local environment rather than direct bactericidal activity. The release of calcium, phosphate, fluoride, strontium, and other ions may suppress cariogenic biofilm activity by reducing environmental acidity and promoting remineralization, thereby creating conditions less favorable for acidogenic microorganisms. This mechanism differs from the direct membrane disruption, ROS generation, or ion‐mediated cytotoxicity typically associated with metallic antibacterial NPs. Although many NP‐based systems demonstrate promising remineralization potential under controlled in vitro conditions, these findings should be interpreted cautiously and not directly extrapolated to long‐term clinical performance without robust in vivo and clinical validation.

**Table 7:** Comparative summary of antibacterial efficacy and mechanical trade‐offs associated with different NP loading ranges in dental composite systems.

NP system	Favorable loading range (wt%)	Antibacterial effect	Mechanical/physical trade‐off at higher loading
ZnO NPs	1–5	Significant reduction in *S. mutans* growth and biofilm formation	Higher concentrations may reduce flexural strength and increase brittleness
TiO_2_ NPs	1–5	Antibacterial and photocatalytic activity with acceptable aesthetics	Excess loading may impair curing depth and increase light scattering
MgO‐NPs	2–5	Effective antibacterial action with pH buffering effects	Higher loading associated with increased porosity and reduced mechanical integrity
AgNPs	0.03–1	Strong broad‐spectrum antibacterial activity	Increased discoloration risk, cytotoxicity concerns, and possible reduction in mechanical stability at higher concentrations
QA‐PEI NPs	0.5–1	Durable contact–active antibacterial effect	Higher concentrations may compromise flexural strength and increase cytotoxicity
Chitosan‐based fillers	≤1	Reduced bacterial adhesion and biofilm formation	Excessive loading may increase water sorption and weaken adhesive/mechanical properties

## 8. Antimicrobial Spectrum and Relevance to Caries Prevention

The oral biofilm constitutes a dynamic and diverse microbial ecosystem; therefore, antibacterial activity against a single microorganism is insufficient to ascertain a material’s potential to prevent caries. *S. mutans* is predominantly utilized for evaluating antibacterial dental nanocomposites due to its role in acid production and extracellular polysaccharide–mediated biofilm formation. Nonetheless, *Streptococcus sobrinus*, *Lactobacillus* spp., *Actinomyces* spp., and complex multispecies microbial communities are also implicated in caries development. Consequently, the antimicrobial efficacy of NP‐modified dental materials should be assessed based on the spectrum of activity and the biological model employed. NPs composed of Ag, ZnO, TiO_2_, and MgO have generally demonstrated effectiveness against *S. mutans*, with some studies also reporting activity against *Lactobacillus* spp., *S. sobrinus*, *E. faecalis*, *S. aureus*, *and C. albicans*. Similarly, materials incorporating QA‐PEI NPs have shown prolonged contact‐active activity against *S. mutans* and, in certain studies, against *Lactobacillus casei*, *E. faecalis*, *S. aureus*, and other microorganisms. Additionally, chitosan‐based NPs and ion‐releasing fillers, such as S‐PRG and BG, may indirectly reduce biofilm formation through surface modification, pH buffering, and the release of ions such as calcium, phosphate, fluoride, and zinc. Although the antimicrobial activity against microorganisms such as *E. faecalis*, *S. aureus*, *and C. albicans* is mainly pertinent to endodontic, prosthodontic, implant, or general antimicrobial uses, it does not serve as direct evidence for caries prevention. More clinically relevant insights are derived from studies involving *S. mutans–Lactobacillus* models, acidogenicity and lactic‐acid assays, and demineralization models of enamel or dentin, with multispecies biofilm studies being particularly informative. Notably, S‐PRG studies utilizing a 39‐species subgingival biofilm model exemplify biologically relevant testing, yet the majority of the evidence remains at the preclinical stage. Table 8 summarizes the antimicrobial spectrum reported for the major NP‐based dental additives and highlights their respective relevance to caries prevention.

**Table 8:** Antimicrobial spectrum of nanoparticle‐based dental additives and their relevance to caries prevention.

Additive group	Main organisms reported	Relevance to caries prevention
Ag, ZnO, TiO_2_, MgO‐NPs	*S. mutans*; selected studies: *Lactobacillus* spp., *E. faecalis*, *S. aureus*, *C. albicans*	Strong laboratory antibacterial evidence; caries relevance is highest where *S. mutans*, acid production, or demineralization are assessed
QA‐PEI NPs	*S. mutans*, *L. casei*, *E. faecalis*, *S. aureus*	Durable contact‐active effects; promising for limiting biofilm at restoration margins
Chitosan‐based NPs	Mainly *S. mutans* and biofilm‐related outcomes	Potential antibacterial and antiadhesive effects; evidence remains limited
Bioactive glass and S‐PRG fillers	Multispecies biofilms and indirect microbial modulation	May support caries prevention through pH buffering and ion release rather than direct killing

## 9. Evaluation Methods for Antibacterial and Bioactive Performance

### 9.1. Methods for Evaluating Bioactivity

The antibacterial properties of NP‐infused dental composites are assessed through various in vitro methodologies. Direct contact assays evaluate bacterial growth in a small volume of broth applied directly to the surfaces of the materials, providing a comprehensive assessment of contact‐mediated bactericidal activity for both leaching and nonleaching systems [179–181]. Agar diffusion assays are utilized to identify zones of inhibition surrounding specimens and are particularly suitable for materials that release soluble agents [182]. Numerous composites containing QA‐PEI and QAC demonstrate minimal or no inhibition zones, thereby confirming their nonleaching properties [183].

**Figure 11:**
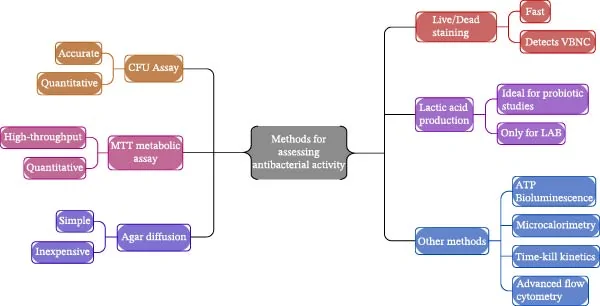
Different methods employed for antibacterial activity assessments.

In quantitative biofilm assays, resin discs are typically incubated with bacterial suspensions under standardized conditions. This is followed by the quantification of CFU, evaluation of metabolic activity using assays such as 3‐(4,5‐dimethylthiazol‐2‐yl)‐2,5‐diphenyltetrazolium bromide (MTT) or XTT, and live/dead cell staining through confocal microscopy [184–187]. These techniques have been extensively applied to assess Ag‐BG composites [13, 56, 188], ZnO NP composites [189–191], chitosan‐based composites [160, 192, 193], and N‐TiO_2_‐containing adhesives [194–196]. In bioactivity assessments, specimens are commonly submerged in SBF or artificial saliva for prolonged durations. Subsequent analyses are conducted using scanning electron microscopy (SEM), energy‐dispersive spectroscopy (EDS), and X‐ray diffraction (XRD) to identify the formation of apatite. Ion release profiles are determined using inductively coupled plasma optical emission spectroscopy, which targets ions such as Ca^2+^, PO_4_^3−^, F^−^, Zn^2+^, Sr^2+^, and Ag^+^, while monitoring pH variations over time. These techniques have been extensively utilized in the study of materials like Ag‐BG composites, S‐PRG composites, Zn‐doped nanoglasses, and phosphate‐based Zn^2+^‐releasing glass liners [197–200]. Figure 11 presents the diverse methodologies employed in evaluating antibacterial activity. In situ investigations using removable intraoral appliances have produced data of clinical significance. In situ investigations using S‐PRG tooth‐coating materials and composites have shown a decrease in enamel slab demineralization and changes in the microbial composition of biofilms formed in volunteers, thereby reinforcing the findings obtained from in vitro studies [36, 48, 201]. Despite this, there is a clear lack of long‐term, randomized clinical trials focused on NP‐containing composites, indicating a considerable gap in the current body of evidence.

### 9.2. In Vivo Evaluation and the Influence of Saliva on Antibacterial Materials

While in vitro antibacterial assays, such as agar diffusion, CFU counting, and metabolic activity tests, are commonly employed to evaluate antibacterial dental materials, they do not adequately replicate the complex conditions present in the oral cavity. The accurate assessment of the antibacterial and bioactive properties of restorative materials requires in vivo clinically relevant evaluation techniques. Within the oral environment, restorative materials are exposed to a dynamic biological milieu, including saliva flow, food particles, PH fluctuations, enzymatic activity, and multispecies microbial biofilm complexes [202]. These environmental factors significantly influence bacterial adhesion, biofilm maturation, and the long‐term efficacy of antibacterial materials. Some studies emphasize the importance of supplementing traditional in vitro assays with in situ or in vivo models to gain a more comprehensive understanding of the clinical performance of antibacterial restorative materials [203, 204]. Saliva plays a crucial role in mediating the interaction between restorative materials and oral microorganisms [205]. Upon placement in the oral cavity, restorative surfaces are rapidly coated with a salivary pellicle composed of proteins, glycoproteins, enzymes, and other biomolecules. This pellicle alters the physicochemical properties of the material surfaces, such as surface free energy, hydrophilicity, and roughness, thereby affecting bacterial adhesion and initial biofilm formation [206, 207]. The adsorption of salivary proteins can either facilitate or inhibit microbial colonization, depending on the pellicle composition and the surface chemistry of the restorative material [208]. Antibacterial materials that demonstrate high activity in simplified laboratory settings may exhibit different responses in the presence of saliva‐derived microcosm biofilms, which more accurately represent the diversity of microbial communities in the oral cavity [209]. The solubility and degradation characteristics of bioactive restorative materials are crucial factors affecting their antibacterial performance [210]. Modern bioactive dental products, such as GICs, BG‐based composites, and CaP‐based fillings, possess antibacterial and remineralizing capabilities due to their controlled ion release. The ions released, including fluoride, calcium, phosphate, zinc, and Ag, can inhibit bacterial activity, increase local pH, and promote the remineralization of demineralized dental tissues [211, 212]. If dissolution is unregulated or solubility is excessive, it can undermine the mechanical integrity and longevity of these restorative materials. Standardized testing systems that evaluate water sorption, solubility, and ion release rates in simulated oral environments are vital for determining long‐term stability [213, 214].

**Figure 12:**
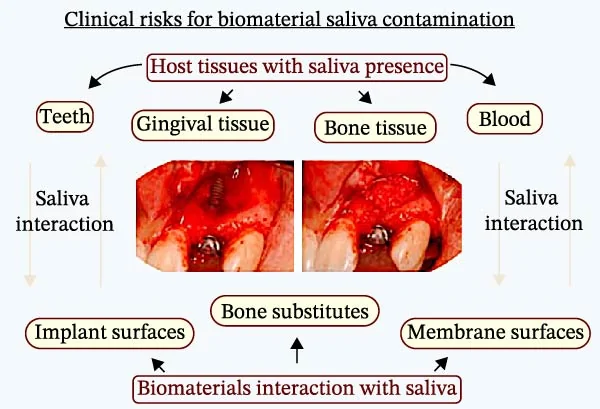
Diagram showing the risk of saliva contamination of biomaterials during oral surgical procedures, where constant exposure leads to early interactions [215] (Under CC License).

Figure 12 depicts the risk of saliva contamination of biomaterials during oral surgical procedures due to constant exposure to the oral environment. Clinical studies indicate that saliva exposure should be carefully controlled to prevent alterations in biomaterials placed in the oral environment; although early contact is not definitively linked to treatment failure, it may compromise properties essential for rapid healing [215]. Salivary esterases and bacterial enzymes can expedite the degradation of resin‐based materials in the oral cavity [216]. These enzymes can break ester bonds in resin matrices and adhesives, leading to surface roughness and reduced strength of the resin–dentin bond [217]. This degradation can facilitate bacterial growth and the onset of secondary caries, thereby compromising the restorations [218, 219]. Thus, extended exposure to saliva and oral biofilms can significantly influence the stability and effectiveness of restorative materials. This combination of factors underscores the importance of combining evaluation methods, including laboratory antibacterial tests, salivary‐derived biofilm normalizations, artificial saliva aging experiments, and in vivo or clinical experiments. These kinds of multidisciplinary strategies can offer a more lifelike clarity of the interactions between antibacterial and bioactive dental materials, saliva, oral microbiota, and host tissues and eventually determine how well they can prevent biofilm formation and secondary caries in clinical practice [207, 220].

## 10. General Trends in the Influence of NPs on Mechanical, Physical, and Aesthetic Properties

This section outlines the overall trends concerning the impact of antibacterial and bioactive NPs on the mechanical, physical, and aesthetic properties of resin‐based dental composites. It does not offer an exhaustive, filler‐specific analysis of all materials examined in this review. NPs have the potential to profoundly affect the core physical properties of composite materials. When these NPs are evenly dispersed and chemically bonded to the matrix, they can enhance the resin by acting as stress‐transfer centers and reducing crack propagation [221, 222]. On the other hand, if they are clumped together or inadequately bonded, they might function as defects [223–225]. Numerous studies have documented that composites incorporating up to 2–3 wt% of well‐silanized ZnO NPs, TiO_2_‐NPs, MgO‐NPs, or QA‐PEI NPs tend to either maintain or slightly enhance their flexural strength and modulus when compared to control samples. In contrast, using higher concentrations often results in diminished strength, modulus, and fracture toughness [5, 7, 47, 125, 141]. CNC/ZnO nanohybrids, tetrapod ZnO whiskers, and specific N‐TiO_2_ formulations have been found to exert substantial reinforcing effects when used at their optimal concentrations [112, 123, 226]. NPs can scatter light, which may reduce light transmission when present in high concentrations or as large agglomerates. This effect can potentially reduce the DC and depth of cure, particularly with opaque fillers such as ZnO and TiO_2_. However, adhesives containing N‐TiO_2_ have demonstrated an improved DC, likely due to better light absorption and a rise in local temperature during polymerization [122, 123, 227]. Hydrophilic NPs, such as chitosan and certain BGs, have the ability to enhance water absorption and solubility, which can potentially accelerate the hydrolytic degradation and plasticization of the resin matrix [228–230]. Investigations into the water sorption and solubility of adhesives containing N‐TiO_2_ indicate that appropriately formulated systems can comply with the limits specified in ISO 4049 for polymer‐based restorative materials. However, composites modified with chitosan require meticulous regulation of particle content to avoid excessive absorption [160, 161, 231]. The attributes of color and translucency are essential for achieving an aesthetically pleasing restoration. Ag and Cu NPs are inherently colored, and even small quantities can cause gray or greenish discoloration if not encapsulated within glassy matrices [55, 232, 233]. In contrast, ZnO, TiO_2_, and MgO‐NPs are white and, when appropriately sized, can be integrated into the filler system without significantly affecting shade matching [234, 235]. N‐TiO_2_ systems have been specifically designed to maintain translucency, and aesthetic studies have generally reported clinically acceptable color differences [123, 125].

Recent in vitro investigations have demonstrated that when inorganic and polymeric nanofillers are well‐dispersed and chemically bonded to the matrix, they function as stress‐transfer centers. This configuration reduces crack propagation and has the potential to enhance flexural strength and modulus at low loadings, typically ≤2–3 wt%. In contrast, higher concentrations of poorly dispersed agglomerates behave as structural flaws and are consistently associated with reduced strength and fracture toughness [143, 236, 237]. In direct resin composites, the incorporation of 1–5 wt% ZnO NPs generally maintained or modestly improved the flexural strength and hardness while providing antibacterial effects. In contrast, loadings ≥ 10 wt% or inadequate silanization tended to decrease the mechanical performance [105, 238]. Similar trends have been reported for MgO‐reinforced bioactive composites: 1–5 wt% MgO yields a favorable balance between flexural properties and antibacterial activity, whereas 10–15 wt% produces brittleness and increased porosity [140, 143]. QA‐PEI NP–modified experimental composites usually retain clinically acceptable flexural strength, modulus, and hardness at about 0.5–1 wt%, but progressive deterioration of mechanical properties is observed as the filler content approaches 3 wt%, paralleling increased cytotoxicity in some formulations [7, 239]. Restorative composites containing chitosan and chitosan/DCPA exhibit a consistent trend, wherein a concentration of ≤0.5–1 wt% typically maintains or slightly improves flexural strength and fracture toughness. In contrast, higher concentrations (≥3–5 wt%) tend to reduce flexural strength and increase microleakage [160, 161]. Polymerization behavior is also influenced by the addition of NPs. Opaque fillers such as ZnO, TiO_2_, and MgO increase light scattering; at high volume fractions or in the presence of large agglomerates, this can reduce light transmission, DC, and depth of cure, especially in bulk‐fill materials [240, 241]. Nevertheless, several N‐TiO_2_ and codoped TiO_2_ systems have demonstrated equal or even higher DC and biaxial flexural strengths compared with unmodified adhesives, an effect attributed to improved light absorption and localized temperature rise during curing. Experimental N‐TiO_2_–containing dental adhesives showed sustained antibacterial activity while maintaining sorption and solubility within ISO 4049 limits, even at NP contents of up to 20 vol% in some systems [123, 125, 126]. By contrast, hydrophilic NPs, such as chitosan and certain mesoporous or phosphate‐based BGs, tend to increase water sorption and solubility, which may accelerate the hydrolytic degradation and plasticization of the resin matrix. Recent systematic and experimental studies emphasize that chitosan should be kept at low concentrations (≤1 wt%) to avoid excessive water uptake and loss of bond strength [242, 243]. Optical properties are particularly critical for aesthetic restoration. Ag‐ and Cu‐containing nanocomposites, even at relatively low loadings, are prone to gray or brown discoloration and increased Δ*E* values after aging. Multiple in vitro studies on AgNP‐modified composites and intracanal medicaments have confirmed noticeable changes in tooth and material color, especially at higher Ag concentrations [244–246]. In contrast, ZnO, TiO_2_, and MgO‐NPs are intrinsically white; when used at optimized sizes and loadings, they can often be incorporated without clinically perceptible shade mismatch and may even improve translucency through more tooth‐like light scattering [126, 160]. Recent work on 3D‐printed and conventional PMMA denture base resins reinforced with TiO_2_ or other oxide nanofillers has reported acceptable color stability, Δ*E*_00_ below common perceptibility thresholds, along with improved surface hardness and wear resistance, provided that the NP content remains in the low‐to‐moderate range ≈ 1–5 wt% [245]. More broadly, spectrophotometric analyses indicate that both filler size distribution and water sorption strongly influence the long‐term color stability of NP‐modified composites [247]. Formulations that limit hygroscopic uptake and maintain a fine, well‐dispersed NP fraction tend to show the smallest color changes in staining solutions [243, 248, 249]. Recent experimental and review data converge on a common design window: Low, well‐silanized NP loadings, typically ≤2–3 wt% for dense inorganic fillers and ~1 wt% for highly active systems such as QA‐PEI or chitosan, can preserve or modestly enhance mechanical properties and depth of cure, while higher concentrations, inadequate surface treatment, and strong hydrophilicity increase the risks of embrittlement, incomplete polymerization, water‐mediated degradation, and aesthetic compromise [250, 251]. Optimizing particle chemistry, morphology, surface functionalization, and total volume fraction is therefore essential to balance antibacterial functions with mechanical integrity and optical performance in NP‐enhanced dental composites.

## 11. Limitations and Future Directions

Although most NP‐containing antibacterial bioactive composites remain in the in vitro or in situ stage, several technologies are closer to, or already in, clinical use [252]. S‐PRG–containing composites and tooth‐coating materials are commercially available and are supported by clinical and in situ data showing reduced WSLs, enhanced enamel microhardness, and modulation of subgingival flora [253]. QAC‐containing adhesives and composites have undergone early clinical and in situ evaluations, where they reduced bacterial counts at restoration margins and decreased enamel demineralization without compromising bond strength [33, 254]. Experimental Ag‐BG composites, ZnO‐ and MgO‐NP composites, QA‐PEI composites, and N‐TiO_2_ adhesives have all demonstrated promising properties in vitro and, in some cases, in animal models but still lack robust long‐term human clinical trials. These materials are likely to be especially beneficial for high‐risk individuals, such as patients with xerostomia, orthodontic appliances, reduced manual dexterity, or systemic conditions, such as uncontrolled diabetes [255, 256]. Major limitations that currently hinder widespread translation include incomplete standardization of antibacterial and bioactivity tests, difficulty in comparing the efficacy of different systems, uncertainty regarding long‐term ion release and NP stability, and unresolved regulatory and environmental issues. Economic considerations, such as the cost of specialized NPs and manufacturing complexity, may also influence adoption [257].

Future research should focus on multifunctional smart nanocomposites that integrate several desirable properties into a single material. Such systems may combine contact‐active QA‐PEI or QAC NPs, ion‐releasing Ag‐ or Zn‐doped BG, fluoride‐containing S‐PRG fillers, and chitosan‐based carriers for peptides or enzymes. In principle, these composites could provide sustained antibacterial effects, remineralization of adjacent tooth tissues, inhibition of MMPs and other host enzymes, and perhaps even controlled release of anti‐inflammatory or regenerative agents. Another promising direction is the development of pH‐responsive or biofilm‐responsive nanocarriers that release antibacterial ions or drugs under acidic or biofilm‐rich conditions, thereby minimizing unnecessary exposure and potential toxicity. Advances in high‐throughput biofilm models, microfluidic platforms, and omics (metagenomics, transcriptomics, and metabolomics) will enable a deeper understanding of how these materials shape the oral microbiome over time [258]. Standardization of the testing protocols is urgently required. Recent critical reviews have called for harmonized in vitro models that simulate dynamic salivary flow, pellicle formation, and multispecies interactions, as well as agreed‐upon outcome measures for antibacterial and remineralizing performance. Only with such standardization will it be possible to rank different NP and monomer systems and compare products meaningfully. Finally, large‐scale, long‐term randomized clinical trials and postmarketing surveillance are necessary to determine whether NP‐containing antibacterial bioactive composites result in a lower incidence of secondary caries and longer restoration survival in real‐world practice. Investigations should focus on groups with heightened risk and define precise endpoints, such as the longevity of dental restorations, the incidence of dental caries, and patient‐reported outcomes.

A key, but sometimes neglected, issue is the potential long‐term effects of antibacterial use in the oral cavity. The majority of antibacterial NP systems target the suppression of cariogenic microorganisms, that is, *S. mutans*, but the oral cavity harbors a complex microbial community that requires a dynamic ecological balance of microbes rather than complete elimination of bacteria to maintain health. Inappropriate and/or prolonged antimicrobial use can negatively impact beneficial commensal species that maintain microbial homeostasis, thereby disrupting biofilm composition and contributing to dysbiosis. Also, prolonged exposure to sublethal doses of antimicrobial ions or ROS could exert selective pressure, favoring the development of more resistant microbial populations. The potential for clinically important resistance development in NP‐based dental materials has not been adequately explored at this time but is not yet proven. Importantly, most of the studies available use models based on a monospecies biofilm or simplified multispecies biofilm models that fail to fully represent the complexity of the oral microbiome. Future studies should also consider microbiome‐level analysis, long‐term biofilm models, and clinical studies to assess the antimicrobial effectiveness and the ecological impact of chronic exposure to antimicrobial agents. The oral microbiome consists of complex and diverse microbial communities that play a role in oral health by balancing and resisting colonization. Although the main purpose of antibacterial NPs is to inhibit cariogenic species, they may not be selective and could affect beneficial commensal microorganisms, which are important for biofilm stability and host defense. These changes can affect the structure of microbial communities, microbial metabolism, and intraspecific and interspecific interactions, with their impacts on the environment largely unknown. A recurring limitation across the current literature is the predominance of laboratory‐based investigations and the lack of standardized methodologies for evaluating antibacterial and bioactive performance. Considerable variation exists in NP synthesis methods, concentrations, particle size distributions, biofilm models, aging protocols, and outcome measures. As a result, direct comparison between studies is often difficult, and the reproducibility of reported findings remains uncertain.

Future studies need to go beyond this to establish antibacterial activity and should include clinically relevant endpoints supporting translational decision making. Beyond determining the ability to inhibit the formation of biofilm and antibacterial activity, studies should also measure the incidence of secondary caries, the survival of the restored tooth after 5 years, the integrity of the margins, the durability of long‐term ion release, the color stability, the wear resistance, and the biological safety under clinically relevant conditions. Further microbiological testing should also include the changes of oral microbial ecology and biofilm composition. In addition, future clinical studies should include patient‐centered outcome measures, such as restoration aesthetics, functional performance, patient satisfaction, and quality of life measures. It is crucial to establish evidence on these endpoints to assess the clinical relevance of the use of antibacterial restorative materials.

## 12. Conclusions

NP‐based antibacterial bioactive composites represent a significant innovation in restorative dentistry. These composites are crafted using a variety of NPs, including metallic NPs such as Ag; metal oxide NPs such as ZnO, TiO_2_, and MgO; polymeric NPs such as QA‐PEI; and organic NPs such as chitosan. Furthermore, they incorporate ion‐releasing BG and S‐PRG fillers, which together exhibit strong antibacterial properties in vitro and, in certain instances, show notable remineralizing and host‐modulating effects. When these NPs are precisely formulated at optimal concentrations, they can be seamlessly integrated into resin composites and adhesives without compromising their mechanical properties, aesthetic appeal, or biocompatibility. Among the current advancements, S‐PRG–containing restorative materials and QAC‐ or QA‐PEI–containing resins are approaching routine clinical use, while Ag‐BG nanocomposites, ZnO‐ and MgO‐NP composites, N‐TiO_2_ adhesives, and chitosan‐hybrid systems are progressing through preclinical and early translational phases. Future materials are anticipated to be multifunctional, incorporating contact killing, ion release, remineralization, and host modulation within a single nanostructured framework. However, several critical challenges remain, including the need for standardized testing protocols, clearly defined regulatory pathways, reliable long‐term safety data, and compelling clinical evidence demonstrating improved outcomes. Addressing these challenges will be crucial for the widespread adoption of NP‐containing antibacterial bioactive composites, which have the potential to significantly reduce secondary caries and extend the lifespan of dental restorations.

## Author Contributions

**Athulya Mullappally:** investigation, data curation, methodology, literature search, visualization, writing – original draft. **Vishnu Vijay Kumar**: conceptualization, writing – original draft, writing – review and edit, supervision. **Arpitha G R**: conceptualization, supervision.

## Funding

This research did not receive any specific grant from funding agencies in the public, commercial, or not‐for‐profit sectors.

## Disclosure

All content was critically reviewed and verified by the authors, who take full responsibility for the accuracy, integrity, and originality of the work.

## Conflicts of Interest

The authors declare no conflicts of interest.

## Data Availability

No new data were generated in this study.

## References

[1] Imazato S. , Antibacterial Properties of Resin Composites and Dentin Bonding Systems, Dental Materials. (2003) 19, no. 6, 449–457, 10.1016/S0109-5641(02)00102-1. 12837391

[2] Zhang J. , Yang Y. , and Chen Y. , et al.A Review of New Generation of Dental Restorative Resin Composites With Antibacterial, Remineralizing and Self-Healing Capabilities, Discover Nano. (2024) 19, no. 1, 10.1186/s11671-024-04151-0, 189. PMC11582236 39570468

[3] Beyth N. , Farah S. , Domb A. J. , and Weiss E. I. , Antibacterial Dental Resin Composites, Reactive and Functional Polymers. (2014) 75, 81–88, 10.1016/j.reactfunctpolym.2013.11.011.

[4] Karunakaran J. , Jayakumar S. , Rajamani S. , Shri B. S. , Nandhini G. , and Srinivasan V. , Revolutionizing Dental Restorations With Nanoparticle-Incorporated Composites, Journal of Conservative Dentistry and Endodontics. (2025) 28, no. 6, 498–504, 10.4103/JCDE.JCDE_98_25. PMC12178550 40546855

[5] Beyth N. , Yudovin-Farber I. , Bahir R. , Domb A. J. , and Weiss E. I. , Antibacterial Activity of Dental Composites Containing Quaternary Ammonium Polyethylenimine Nanoparticles Against *Streptococcus mutans* , Biomaterials. (2006) 27, no. 21, 3995–4002, 10.1016/j.biomaterials.2006.03.003. 16564083

[6] Alansy A. S. , Saeed T. A. , Guo Y. , Yang Y. , Liu B. , and Fan Z. , Antibacterial Dental Resin Composites: A Narrative Review, Open Journal of Stomatology. (2022) 12, no. 5, 147–165, 10.4236/ojst.2022.125015.

[7] Chladek G. , Barszczewska-Rybarek I. , Chrószcz-Porębska M. , and Mertas A. , The Effect of Quaternary Ammonium Polyethylenimine Nanoparticles on Bacterial Adherence, Cytotoxicity, and Physical and Mechanical Properties of Experimental Dental Composites, Scientific Reports. (2023) 13, no. 1, 10.1038/s41598-023-43851-y, 17497. PMC10577145 37840040

[8] Chrószcz M. and Barszczewska-Rybarek I. , Nanoparticles of Quaternary Ammonium Polyethylenimine Derivatives for Application in Dental Materials, Polymers. (2020) 12, no. 11, 10.3390/polym12112551, 2551. PMC7693368 33143324

[9] Kumar V. V. , Gara D. , Subramanian S. , Vijayan M. , Ramakrishna S. , and Chandran S. , Lightweight Foams for Biomedical Applications, Lightweight Composites, 2026, Elsevier, 383–419.

[10] Kumar V. V. , Effect of Hybridization and Nanofiber Interleaving on the Mechanical Properties of Fiber Reinforced Polymer Composites, 2023, National University of Singapore (Singapore).

[11] Chatzistavrou X. , Velamakanni S. , and DiRenzo K. , et al.Designing Dental Composites With Bioactive and Bactericidal Properties, Materials Science & Engineering. C, Materials for Biological Applications. (2015) 52, 267–272, 10.1016/j.msec.2015.03.062. 25953567

[12] Wang Y.-Y. , Chatzistavrou X. , and Faulk D. , et al.Biological and Bactericidal Properties of Ag-Doped Bioactive Glass in a Natural Extracellular Matrix Hydrogel With Potential Application in Dentistry, European Cells and Materials. (2015) 29, 342–355, 10.22203/eCM.v029a26. 26091732

[13] Kattan H. , Chatzistavrou X. , Boynton J. , Dennison J. , Yaman P. , and Papagerakis P. , Physical Properties of an Ag-Doped Bioactive Flowable Composite Resin, Materials. (2015) 8, no. 8, 4668–4678, 10.3390/ma8084668. PMC5455494 28793463

[14] Wang W. , Chen R. , and Xie Y. , et al.Research Progress of Bioactive Glass in the Remineralization of Dental Hard Tissue, Biomedical Physics & Engineering Express. (2025) 11, no. 5, 10.1088/2057-1976/adfb81, 052001. 40812320

[15] Aryah U. , Prasanth N. , Stani R. , Kandanattu B. , and George N. , Dental Disease Association With Bacterial Biofilm, Bacterial Biofilm and Chronic Infections: Role in Disease Pathogenesis and Therapeutic Strategies, 2025, Springer, 99–134.

[16] Goldberg M. , Understanding Dental Caries, 2016, Springer.

[17] Walsh L. J. , Dental Plaque Fermentation and Its Role in Caries Risk Assessment, International Dentistry SA. (2006) 8, no. 5, 34–40.

[18] Soro A. S. , Lamont R. J. , Egland P. G. , Koo H. , and Liu Y. , Dental Caries, Molecular Medical Microbiology, 2024, Elsevier, 915–930, 10.1016/B978-0-12-818619-0.00036-8.

[19] Barani-Sveçla M. and Buleshkaj S. , Etiopathogenesis of Dental Caries, Enamel and Dentin-Pulp Complex, 2024, IntechOpen.

[20] Abou Neel E. , Aljabo A. , and Strange A. , et al.Demineralization–Remineralization Dynamics in Teeth and Bone, International Journal of Nanomedicine. (2016) 11, 4743–4763, 10.2147/IJN.S107624. PMC5034904 27695330

[21] Dudás C. , Kardos E. , and Székely M. , et al.Effect of Glass Fiber Reinforcement on Marginal Microleakage in Class II Composite Restorations: An In Vitro Pilot Study, Dentistry Journal. (2024) 12, no. 12, 10.3390/dj12120410, 410. PMC11674346 39727467

[22] Brambilla E. and Ionescu A. C. , Oral Biofilms and Secondary Caries Formation, Oral Biofilms and Modern Dental Materials: Advances Toward Bioactivity, 2021, Springer, 19–35.

[23] Zhou W. , Peng X. , and Zhou X. , et al.Novel Nanocomposite Inhibiting Caries at the Enamel Restoration Margins in an In Vitro Saliva-Derived Biofilm Secondary Caries Model, International Journal of Molecular Sciences. (2020) 21, no. 17, 10.3390/ijms21176369, 6369. PMC7503730 32887330

[24] Kumar V. V. , Shiva S. , and Asuwin Prabu R. G. , et al.Recent Advances in Additive Manufacturing for Printing Biological Tissues, Bioactive Glass Materials for Biological Applications, 2025, 1st edition, CRC Press, 136–152, 10.1201/9781003660781-7.

[25] Yaghmoor R. B. , In Vitro Evaluation of Novel Antibacterial Dental Composites, 2022, UCL (University College London).

[26] Parhi S. , Pal S. , Das S. K. , and Ghosh P. , Strategies Toward Development of Antimicrobial Biomaterials for Dental Healthcare Applications, Biotechnology and Bioengineering. (2021) 118, no. 12, 4590–4622, 10.1002/bit.27948. 34599764

[27] Bignozzi I. , Crea A. , Capri D. , Littarru C. , Lajolo C. , and Tatakis D. N. , Root Caries: A Periodontal Perspective, Journal of Periodontal Research. (2014) 49, no. 2, 143–163, 10.1111/jre.12094. 23647556

[28] Song F. , Koo H. , and Ren D. , Effects of Material Properties on Bacterial Adhesion and Biofilm Formation, Journal of Dental Research. (2015) 94, no. 8, 1027–1034, 10.1177/0022034515587690. 26001706

[29] Barjandi G. , Svedenlöf J. , and Jasim H. , et al.Clinical Aspects of Mastication Myalgia—An Overview, Frontiers in Pain Research. (2024) 4, 10.3389/fpain.2023.1306475, 1306475. PMC10803665 38264542

[30] Dutra D. A. M. , Pereira G. K. R. , Kantorski K. Z. , Valandro L. F. , and Zanatta F. B. , Does Finishing and Polishing of Restorative Materials Affect Bacterial Adhesion and Biofilm Formation? A Systematic Review, Operative Dentistry. (2018) 43, no. 1, E37–E52, 10.2341/17-073-L. 29284102

[31] Bollenl C. M. L. , Lambrechts P. , and Quirynen M. , Comparison of Surface Roughness of Oral Hard Materials to the Threshold Surface Roughness for Bacterial Plaque Retention: A Review of the Literature, Dental Materials. (1997) 13, no. 4, 258–269, 10.1016/S0109-5641(97)80038-3. 11696906

[32] Ferracane J. L. , Resin Composite—State of the Art, Dental Materials. (2011) 27, no. 1, 29–38, 10.1016/j.dental.2010.10.020. 21093034

[33] Melo M. A. S. , Garcia I. M. , and Alluhaidan T. , et al.The Next Frontier in Antibacterial Dental Resins: A 20-Year Journey of Innovation and Expectations, Dental Materials. (2025) 41, no. 9, 1045–1057, 10.1016/j.dental.2025.06.013. 40527702

[34] Sun Q. , Zhang L. , and Bai R. , et al.Recent Progress in Antimicrobial Strategies for Resin-Based Restoratives, Polymers. (2021) 13, no. 10, 10.3390/polym13101590, 1590. PMC8156482 34069312

[35] Miki S. , Kitagawa H. , Kitagawa R. , Kiba W. , Hayashi M. , and Imazato S. , Antibacterial Activity of Resin Composites Containing Surface Pre-Reacted Glass-Ionomer (S-PRG) Filler, Dental Materials. (2016) 32, no. 9, 1095–1102, 10.1016/j.dental.2016.06.018. 27417376

[36] de Lima G. C. , de Cassia Orlando Sardi J. , and de Figueiredo L. C. , et al.Antibacterial Activity of a Bioactive Composite Resins Containing Surface Pre-Reacted Glass in a Complex Multispecies Subgingival Biofilm, Odontology. (2025) 114, no. 2, 1–11. 10.1007/s10266-025-01153-x 40676445

[37] Choi Y. , Sun W. , and Kim Y. , et al.Effects of Zn-Doped Mesoporous Bioactive Glass Nanoparticles in Etch-and-Rinse Adhesive on the Microtensile Bond Strength, Nanomaterials. (2020) 10, no. 10, 10.3390/nano10101943, 1943. PMC7601785 33003534

[38] Deng F. , Kitagawa H. , and Kohno T. , et al.Fabrication of Rapidly Soluble Zn^2+^-Releasing Phosphate-Based Glass and Its Incorporation Into Dental Resin, Molecules. (2024) 29, no. 21, 10.3390/molecules29215098, 5098. PMC11547867 39519739

[39] Bhattacharjee B. , Ghosh S. , Patra D. , and Haldar J. , Advancements in Release-Active Antimicrobial Biomaterials: A Journey From Release to Relief, WIREs Nanomedicine and Nanobiotechnology. (2022) 14, no. 1, 10.1002/wnan.1745. 34374498

[40] Ramburrun P. , Pringle N. A. , Dube A. , Adam R. Z. , D’Souza S. , and Aucamp M. , Recent Advances in the Development of Antimicrobial and Antifouling Biocompatible Materials for Dental Applications, Materials. (2021) 14, no. 12, 10.3390/ma14123167, 3167. PMC8229368 34207552

[41] Roy S. , Anti-Microbial Activities of Nano-Polymers for Biomedical Applications, Assessment of Polymeric Materials for Biomedical Applications, 2023, CRC Press, 101–120.

[42] Bhadila G. Y. , Novel Dental Nanocomposites With Low-Shrinkage-Stress, Ion Recharge, Antibacterial and Remineralization Capabilities to Protect Tooth Structures, 2021, University of Maryland.

[43] Andersen C. , Madsen J. , and Daugaard A. E. , A Synthetic Overview of Preparation Protocols of Nonmetallic, Contact-Active Antimicrobial Quaternary Surfaces on Polymer Substrates, Macromolecular Rapid Communications. (2021) 42, no. 21, 10.1002/marc.202100437, 2100437. 34491589

[44] Loontjens J. , Quaternary Ammonium Compounds, Biomaterials Associated Infection: Immunological Aspects and Antimicrobial Strategies, 2012, Springer, 379–404.

[45] Akbarzadeh F. Z. , Sarraf M. , and Ghomi E. R. , et al.A State-of-the-Art Review on Recent Advances in the Fabrication and Characteristics of Magnesium-Based Alloys in Biomedical Applications, Journal of Magnesium and Alloys. (2024) 12, no. 7, 2569–2594, 10.1016/j.jma.2024.06.015.

[46] Al-Mosawi R. M. and Al-Badr R. M. , The Study Effects of Dental Composite Resin as Antibacterial Agent which Contain Nanoparticles of Zinc Oxide on the Bacteria Associated With Oral Infection, IOSR Journal of Dental and Medical Sciences. (2017) 16, no. 1, 49–55, 10.9790/0853-1601014955.

[47] Arif W. , Rana N. , and Saleem I. , et al.Antibacterial Activity of Dental Composite With Ciprofloxacin Loaded Silver Nanoparticles, Molecules. (2022) 27, no. 21, 10.3390/molecules27217182, 7182. PMC9658858 36364007

[48] Mayumi K. , Miyaji H. , and Miyata S. , et al.Antibacterial Coating of Tooth Surface With Ion-Releasing Pre-Reacted Glass-Ionomer (S-PRG) Nanofillers, Heliyon. (2021) 7, no. 2, 10.1016/j.heliyon.2021.e06147. PMC7889979 33644453

[49] Poletto P. S. , Marin M. N. , Brancini M. L. , Soffener T. , de Araújo B. , and dos Reis A. C. , Effect of S-PRG Particles on Demineralization and Remineralization of Dentin: A Clinical Case Report, MedNEXT Journal of Medical and Health Sciences. (2022) 3, no. S6, 10.54448/mdnt22S606, 6.

[50] Saverina E. A. , Frolov N. A. , Kamanina O. A. , Arlyapov V. A. , Vereshchagin A. N. , and Ananikov V. P. , From Antibacterial to Antibiofilm Targeting: An Emerging Paradigm Shift in the Development of Quaternary Ammonium Compounds (QACs), ACS Infectious Diseases. (2023) 9, no. 3, 394–422, 10.1021/acsinfecdis.2c00469. 36790073

[51] Santoro O. and Izzo L. , Antimicrobial Polymer Surfaces Containing Quaternary Ammonium Centers (QACs): Synthesis and Mechanism of Action, International Journal of Molecular Sciences. (2024) 25, no. 14, 10.3390/ijms25147587, 7587. PMC11277267 39062830

[52] Domínguez A. V. , Algaba R. A. , Canturri A. M. , Villodres Á.R. , and Smani Y. , Antibacterial Activity of Colloidal Silver Against Gram-Negative and Gram-Positive Bacteria, Antibiotics. (2020) 9, no. 1, 10.3390/antibiotics9010036, 36. PMC7167925 31963769

[53] Abbas R. , Luo J. , and Qi X. , et al.Silver Nanoparticles: Synthesis, Structure, Properties and Applications, Nanomaterials. (2024) 14, no. 17, 10.3390/nano14171425, 1425. PMC11397261 39269087

[54] Xu Z. , Zhang C. , Wang X. , and Liu D. , Release Strategies of Silver Ions From Materials for Bacterial Killing, ACS Applied Bio Materials. (2021) 4, no. 5, 3985–3999, 10.1021/acsabm.0c01485. 35006818

[55] Azarsina M. , Kasraei S. , Yousefi-Mashouf R. , Dehghani N. , and Shirinzad M. , The Antibacterial Properties of Composite Resin Containing Nanosilver Against *Streptococcus mutans* and Lactobacillus, The Journal of Contemporary Dental Practice. (2014) 14, no. 6, 1014–1018, 10.5005/jp-journals-10024-1442. 24858742

[56] Zalega M. and Bociong K. , Antibacterial Agents Used in Modifications of Dental Resin Composites: A Systematic Review, Applied Sciences. (2024) 14, no. 9, 10.3390/app14093710, 3710.

[57] Qiang W. P. , He X. D. , and Zhang K. , et al.Mussel Adhesive Mimetic Silk Sericin Prepared by Enzymatic Oxidation for the Construction of Antibacterial Coatings, ACS Biomaterials Science & Engineering. (2021) 7, no. 7, 3379–3388, 10.1021/acsbiomaterials.1c00271. 34161086

[58] Guo Y. , Hou X. , Fan S. , and Jin C. , Research Status of Silver Nanoparticles for Dental Applications, Inorganics. (2025) 13, no. 5, 10.3390/inorganics13050168, 168.

[59] Samiei M. , Aghazadeh M. , Lotfi M. , Shakoei S. , Aghazadeh Z. , and Pakdel S. M. V. , Antimicrobial Efficacy of Mineral Trioxide Aggregate With and Without Silver Nanoparticles, Iranian Endodontic Journal. (2013) 8, no. 4, 166–170, 10.1016/S0099-2399(06)80967-2. PMC3808675 24171023

[60] Lotfi M. , Vosoughhosseini S. , Ranjkesh B. , Khani S. , Saghiri M. , and Zand V. , Antimicrobial Efficacy of Nanosilver, Sodium Hypochlorite and Chlorhexidine Gluconate Against *Enterococcus faecalis* , African Journal of Biotechnology. (2011) 10, no. 35, 6799–6803.

[61] Nam K.-Y. , *In Vitro* Antimicrobial Effect of the Tissue Conditioner Containing Silver Nanoparticles, The Journal of Advanced Prosthodontics. (2011) 3, no. 1, 20–24, 10.4047/jap.2011.3.1.20. PMC3076569 21503189

[62] Flores C. Y. , Diaz C. , and Rubert A. , et al.Spontaneous Adsorption of Silver Nanoparticles on Ti/TiO_2_ Surfaces. Antibacterial Effect on *Pseudomonas aeruginosa* , Journal of Colloid and Interface Science. (2010) 350, no. 2, 402–408, 10.1016/j.jcis.2010.06.052. 20656295

[63] Zhao L. , Wang H. , and Huo K. , et al.Antibacterial Nano-Structured Titania Coating Incorporated With Silver Nanoparticles, Biomaterials. (2011) 32, no. 24, 5706–5716, 10.1016/j.biomaterials.2011.04.040. 21565401

[64] Cheng L. , Weir M. D. , and Xu H. H. K. , et al.Antibacterial Amorphous Calcium Phosphate Nanocomposites With a Quaternary Ammonium Dimethacrylate and Silver Nanoparticles, Dental Materials. (2012) 28, no. 5, 561–572, 10.1016/j.dental.2012.01.005. PMC3322309 22305716

[65] Li F. , Weir M. D. , Chen J. , and Xu H. H. K. , Comparison of Quaternary Ammonium-Containing With Nano-Silver-Containing Adhesive in Antibacterial Properties and Cytotoxicity, Dental Materials. (2013) 29, no. 4, 450–461, 10.1016/j.dental.2013.01.012. PMC3631003 23428077

[66] Melo M. A. S. , Cheng L. , Zhang K. , Weir M. D. , Rodrigues L. K. A. , and Xu H. H. K. , Novel Dental Adhesives Containing Nanoparticles of Silver and Amorphous Calcium Phosphate, Dental Materials. (2013) 29, no. 2, 199–210, 10.1016/j.dental.2012.10.005. PMC3552134 23138046

[67] Zhang K. , Li F. , and Imazato S. , et al.Dual Antibacterial Agents of Nano-Silver and 12-Methacryloyloxydodecylpyridinium Bromide in Dental Adhesive to Inhibit Caries, Journal of Biomedical Materials Research Part B: Applied Biomaterials. (2013) 101B, no. 6, 929–938, 10.1002/jbm.b.32898. PMC3710541 23529901

[68] Farahani A. , Beyrami A. , Piri H. , Naghizadeh A. , Imani H. , and Farahani M. , Evaluation of Antibacterial Properties of Resin Composites Containing Silver Nanoparticles on *Streptococcus mutans* , Journal Dental Oral Health. (2018) 5, 1–6.

[69] Acosta-Torres L. S. , Mendieta I. , Nuñez-Anita R. E. , Cajero-Juárez M. , and Castaño V. M. , Cytocompatible Antifungal Acrylic Resin Containing Silver Nanoparticles for Dentures, International Journal of Nanomedicine. (2012) 7, 4777–4786, 10.2147/IJN.S32391. PMC3435119 22969297

[70] Monteiro D. R. , Gorup L. F. , Takamiya A. S. , de Camargo E. R. , Filho A. C. R. , and Barbosa D. B. , Silver Distribution and Release From an Antimicrobial Denture Base Resin Containing Silver Colloidal Nanoparticles, Journal of Prosthodontics: Implant, Esthetic and Reconstructive Dentistry. (2012) 21, no. 1, 7–15, 10.1111/j.1532-849X.2011.00772.x. 22050139

[71] Arslan S. , Ekrikaya S. , Ildiz N. , Yusufbeyoglu S. , and Ocsoy İ. , Evaluation of the Antibacterial Activity of Dental Adhesive Containing Biogenic Silver Nanoparticles Decorated Nanographene Oxide Nanocomposites (Ag@ nGO NCs) and Effect on Bond Strength to Dentine, Odontology. (2024) 112, no. 2, 341–354, 10.1007/s10266-023-00836-7. 37436660

[72] Abar E. S. , Vandghanooni S. , Memar M. Y. , Eskandani M. , and Torab A. , Enhancing Antifungal and Antibacterial Properties of Denture Resins With Nystatin-Coated Silver Nanoparticles, Scientific Reports. (2024) 14, no. 1, 10.1038/s41598-024-74465-7, 23770. PMC11467417 39390054

[73] Almeida N. L. M. , Peralta L. C. F. , Pontes F. M. L. , Rinaldo D. , Porto V. C. , and Lara V. S. , Anti-Candida Activity and Biocompatibility of Silver Nanoparticles Associated With Denture Glaze: A New Approach to the Management of Denture Stomatitis, Folia Microbiologica. (2024) 69, no. 6, 1229–1246, 10.1007/s12223-024-01161-4. 38652435

[74] Salaie R. N. , Hassan P. A. , Meran Z. D. , and Hamad S. A. , Antibacterial Activity of Dissolved Silver Fractions Released From Silver-Coated Titanium Dental Implant Abutments: A Study on *Streptococcus mutans* Biofilm Formation, Antibiotics. (2023) 12, no. 7, 10.3390/antibiotics12071097, 1097. PMC10376167 37508193

[75] Paiwand S. , Schäfer S. , and Kopp A. , et al.Antibacterial Potential of Silver and Zinc Loaded Plasma-Electrolytic Oxidation Coatings for Dental Titanium Implants, International Journal of Implant Dentistry. (2025) 11, no. 1, 10.1186/s40729-025-00595-w, 12. PMC11833008 39960576

[76] Yoon M. S. , Lee J. M. , and Jo M. J. , et al.Dual-Drug Delivery Systems Using Hydrogel–Nanoparticle Composites: Recent Advances and Key Applications, Gels. (2025) 11, no. 7, 10.3390/gels11070520, 520. PMC12294678 40710682

[77] Harun-Ur-Rashid M. , Foyez T. , Krishna S. B. N. , Poda S. , and Imran A. B. , Recent Advances of Silver Nanoparticle-Based Polymer Nanocomposites for Biomedical Applications, RSC Advances. (2025) 15, no. 11, 8480–8505, 10.1039/D4RA08220F. PMC11920860 40109922

[78] Susanto B. , Putro A. J. N. , and Ristyawan M. R. , et al.Enhanced Mechanical Properties of the Additively Manufactured Modified Hybrid Stereolithography (SLA)–Glass Powder, Journal of Composites Science. (2025) 9, no. 5, 10.3390/jcs9050205, 205.

[79] Xu V. W. , Nizami M. Z. I. , Yin I. X. , Yu O. Y. , Lung C. Y. K. , and Chu C. H. , Application of Copper Nanoparticles in Dentistry, Nanomaterials. (2022) 12, no. 5, 10.3390/nano12050805, 805. PMC8912653 35269293

[80] Eshed M. , Lellouche J. , Matalon S. , Gedanken A. , and Banin E. , Sonochemical Coatings of ZnO and CuO Nanoparticles Inhibit *Streptococcus mutans* Biofilm Formation on Teeth Model, Langmuir. (2012) 28, no. 33, 12288–12295, 10.1021/la301432a. 22830392

[81] Mlinarić N. M. , Zore A. , and Veselinovic V. , et al.Antimicrobial Activity of Poly (methyl methacrylate) Doped With CuO and ZnO Nanoparticles, ACS Omega. (2025) 10, no. 13, 13060–13072, 10.1021/acsomega.4c10170. PMC11983175 40224428

[82] Nikanjam S. , Yeganegi A. , Alikhani M.-Y. , Farmany A. , Ghiasian S. A. , and Hasanzade R. , Novel Antimicrobial Applications of Copper Oxide Nanoparticles After Combination With Tissue Conditioner Used in Complete Prostheses, BMC Oral Health. (2024) 24, no. 1, 10.1186/s12903-024-04534-w, 752. PMC11214236 38943115

[83] Marovic D. , Haugen H. J. , and Mandic V. N. , et al.Incorporation of Copper-Doped Mesoporous Bioactive Glass Nanospheres in Experimental Dental Composites: Chemical and Mechanical Characterization, Materials. (2021) 14, no. 10, 10.3390/ma14102611, 2611. PMC8156461 34067788

[84] Martin R. R. , Naftel S. J. , Nelson A. J. , Edwards M. , Mithoowani H. , and Stakiw J. , Synchrotron Radiation Analysis of Possible Correlations Between Metal Status in Human Cementum and Periodontal Disease, Journal of Synchrotron Radiation. (2010) 17, no. 2, 263–267, 10.1107/S0909049509052807. 20157281

[85] Dannan A. and Hanno Y. , The Relationship Between Periodontal Diseases and Plasma Level of Copper and Magnesium, Dentistry. (2018) 8, no. 2, 10.4172/2161-1122.1000471, 10.4172.

[86] Aguilar-Perez D. , Vargas-Coronado R. , and Cervantes-UC J. M. , et al.Antibacterial Activity of a Glass Ionomer Cement Doped With Copper Nanoparticles, Dental Materials Journal. (2020) 39, no. 3, 389–396, 10.4012/dmj.2019-046. 32213765

[87] Sabatini C. , Mennito A. S. , Wolf B. J. , Pashley D. H. , and Renné W. G. , Incorporation of Bactericidal Poly-Acrylic Acid Modified Copper Iodide Particles Into Adhesive Resins, Journal of Dentistry. (2015) 43, no. 5, 546–555, 10.1016/j.jdent.2015.02.012. PMC5788176 25731156

[88] Hameed H. A. , Ariffin A. , Luddin N. , and Husein A. , Evaluation of Antibacterial Properties of Copper Nanoparticles Surface Coating on Titanium Dental Implant, Journal of Pharmaceutical Sciences and Research. (2018) 10, no. 5, 1157–1160.

[89] Gutiérrez M. F. , Malaquias P. , and Hass V. , et al.The Role of Copper Nanoparticles in an Etch-and-Rinse Adhesive on Antimicrobial Activity, Mechanical Properties and the Durability of Resin-Dentine Interfaces, Journal of Dentistry. (2017) 61, 12–20, 10.1016/j.jdent.2017.04.007. 28438559

[90] Zhang F. , Zhou M. , and Gu W. , et al.Zinc-/Copper-Substituted Dicalcium Silicate Cement: Advanced Biomaterials With Enhanced Osteogenesis and Long-Term Antibacterial Properties, Journal of Materials Chemistry B. (2020) 8, no. 5, 1060–1070, 10.1039/C9TB02691F. 31939984

[91] Gutiérrez M. F. , Alegría-Acevedo L. F. , and Bermudez Lán Méndez-Bauer J. , et al.Biological, Mechanical and Adhesive Properties of Universal Adhesives Containing Zinc and Copper Nanoparticles, Journal of Dentistry. (2019) 82, 45–55, 10.1016/j.jdent.2019.01.012. 30738850

[92] Lin Z. , Cao Y. , and Zou J. , et al.Improved Osteogenesis and Angiogenesis of a Novel Copper Ions Doped Calcium Phosphate Cement, Materials Science and Engineering C. (2020) 114, 10.1016/j.msec.2020.111032, 111032. 32993975

[93] Zhang W. , Chang Q. , and Xu L. , et al.Graphene Oxide-Copper Nanocomposite-Coated Porous CaP Scaffold for Vascularized Bone Regeneration via Activation of Hif-1α, Advanced Healthcare Materials. (2016) 5, no. 11, 1299–1309, 10.1002/adhm.201500824. 26945787

[94] Shoeib A. , Shaker M. , Abdeen R. , and El-Segai A. , Evaluation of Silver and Copper Oxide NANOPARTICLES Release From Different Prosthetic Materials Incorporated With Nanoparticles, Egyptian Dental Journal. (2019) 65, no. 3, 2849–2855, 10.21608/edj.2019.72681.

[95] Bourgi R. , Doumandji Z. , and Cuevas-Suárez C. E. , et al.Exploring the Role of Nanoparticles in Dental Materials: A Comprehensive Review, Coatings. (2025) 15, no. 1, 10.3390/coatings15010033, 33.

[96] Jongrungsomran S. , Pissuwan D. , Yavirach A. , Rungsiyakull C. , and Rungsiyakull P. , The Integration of Gold Nanoparticles Into Dental Biomaterials as a Novel Approach for Clinical Advancement: A Narrative Review, Journal of Functional Biomaterials. (2024) 15, no. 10, 10.3390/jfb15100291, 291. PMC11508227 39452589

[97] Zhu H. , Tan J. , and Qiu J. , et al.Gold Nanoparticles Decorated Titanium Oxide Nanotubes With Enhanced Antibacterial Activity Driven by Photocatalytic Memory Effect, Coatings. (2022) 12, no. 9, 10.3390/coatings12091351, 1351.

[98] Zheng X. , Sun J. , and Li W. , et al.Engineering Nanotubular Titania With Gold Nanoparticles for Antibiofilm Enhancement and Soft Tissue Healing Promotion, Journal of Electroanalytical Chemistry. (2020) 871, 10.1016/j.jelechem.2020.114362, 114362.

[99] Su C. , Huang K. , Li H.-H. , Lu Y.-G. , and Zheng D.-L. , Antibacterial Properties of Functionalized Gold Nanoparticles and Their Application in Oral Biology, Journal of Nanomaterials. (2020) 2020, no. 1, 13, 10.1155/2020/5616379, 5616379.

[100] Moon K.-S. , Park Y.-B. , Bae J.-M. , and Oh S. , Near-Infrared Laser-Mediated Drug Release and Antibacterial Activity of Gold Nanorod–sputtered Titania Nanotubes, Journal of Tissue Engineering. (2018) 9, 10.1177/2041731418790315, 2041731418790315. PMC6071157 30083309

[101] Ivanovic V. , Popovic D. , and Petrovic S. , et al.Unraveling the Antibiofilm Activity of a New Nanogold Resin for Dentures and Epithesis, Pharmaceutics. (2022) 14, no. 7, 10.3390/pharmaceutics14071513, 1513. PMC9322197 35890413

[102] Marić I. , Zore A. , and Rojko F. , et al.Antifungal Effect of Polymethyl Methacrylate Resin Base With Embedded Au Nanoparticles, Nanomaterials. (2023) 13, no. 14, 10.3390/nano13142128, 2128. PMC10383817 37513139

[103] Zhang M. , Liu X. , and Xie Y. , et al.Biological Safe Gold Nanoparticle-Modified Dental Aligner Prevents the *Porphyromonas gingivalis* Biofilm Formation, ACS Omega. (2020) 5, no. 30, 18685–18692, 10.1021/acsomega.0c01532. PMC7407536 32775870

[104] Łyczek J. , Bończak B. , Krzymińska I. , Giżyński K. , and Paczesny J. , Gold–Oxoborate Nanocomposite-Coated Orthodontic Brackets Gain Antibacterial Properties While Remaining Safe for Eukaryotic Cells, Journal of Biomedical Materials Research Part B: Applied Biomaterials. (2023) 111, no. 5, 996–1004, 10.1002/jbm.b.35208. 36462180

[105] Wang Y. , Hua H. , Li W. , Wang R. , Jiang X. , and Zhu M. , Strong Antibacterial Dental Resin Composites Containing Cellulose Nanocrystal/Zinc Oxide Nanohybrids, Journal of Dentistry. (2019) 80, 23–29, 10.1016/j.jdent.2018.11.002. 30423354

[106] Garcia P. P. N. S. , Cardia M. F. B. , and Francisconi R. S. , et al.Antibacterial Activity of Glass Ionomer Cement Modified by Zinc Oxide Nanoparticles, Microscopy Research and Technique. (2017) 80, no. 5, 456–461. 10.1002/jemt.22814 27935662

[107] Quezada-Morales P. D. , Luengo-Machuca L. , and Quezada-Aguiluz M. , et al.Antibacterial Activity of Zinc Oxide Nanoparticles in Self-Curing Acrylic Resin Against *Streptococcus mutans* , International Journal of Odontostomatology. (2021) 15, no. 3, 694–701, 10.4067/S0718-381X2021000300694.

[108] El Shahawi A. M. , Incorporation of Zinc Oxide Nanoparticles and It’s Antibacterial Effect on Toothpaste, Bulletin of the National Research Centre. (2023) 47, no. 1, 10.1186/s42269-022-00975-x, 2.

[109] Albuquerque L. A. , Sangappa S. B. , Srinivasan A. , Archer A. , and Rao R. , Efficacy of Chemically and Biologically Synthesized Zinc Oxide Nanoparticles Incorporated in Soft Denture Liner Against *candida albicans*: A Comparative In Vitro Study, World Journal of Dentistry. (2023) 14, no. 10, 851–859, 10.5005/jp-journals-10015-2311.

[110] Mazur M. W. , Grudniak A. , and Szałaj U. , et al.Antifungal Activity of Newly Formed Polymethylmethacrylate (PMMA) Modification by Zinc Oxide and Zinc Oxide–Silver Hybrid Nanoparticles, Polymers. (2024) 16, no. 24, 10.3390/polym16243512, 3512. PMC11677632 39771364

[111] Zhou S. , Liu R. , and Ma X. , et al.Antibacterial Effect of Novel Dental Resin Composites Containing Rod-Like Zinc Oxide, Nanotechnology Reviews. (2024) 13, no. 1, 10.1515/ntrev-2023-0195, 20230195.

[112] Lee H. , Kim Y.-J. , Yang Y.-J. , Lee J.-H. , and Lee H.-H. , Development of Antibacterial Dual-Cure Dental Resin Composites via Tetrapod-Shaped Zinc Oxide Incorporation, Dental Materials. (2024) 40, no. 11, 1762–1772, 10.1016/j.dental.2024.07.021. 39117497

[113] Zhou J. , Wang H. , and Virtanen S. , et al.Hybrid Zinc Oxide Nanocoating on Titanium Implants: Controlled Drug Release for Enhanced Antibacterial and Osteogenic Performance in Infectious Conditions, Acta Biomaterialia. (2024) 189, 589–604, 10.1016/j.actbio.2024.09.039. 39343288

[114] Bazkiayi A. A. , Amdjadi P. , and Yadegari Z. , et al.Antibacterial and Physicochemical Properties of a Zinc Oxide Nanoparticle-Modified Orthodontic Adhesive: An In Vitro Study, Journal of Orofacial Orthopedics = Fortschritte der Kieferorthopadie: Organ/Official Journal Deutsche Gesellschaft fur Kieferorthopadie. (2025) 87, no. 5, 529–543, 10.1007/s00056-025-00603-z. 40762800

[115] Tariq K. , Rana N. F. , Javaid S. , and Khadim M. , Titanium Surface Functionalization With Calcium-Doped ZnO Nanoparticles for Hard Tissue Implant Applications, SLAS Technology. (2025) 33, 10.1016/j.slast.2025.100337, 100337. 40738248

[116] Pushpalatha J. S. , Gayathri V. S. , and Sowmya S. V. , et al.Zinc Oxide Nanoparticles: A Review on Its Applications in Dentistry, Frontiers in Bioengineering and Biotechnology. (2022) 10, 10.3389/fbioe.2022.917990, 917990. PMC9160914 35662838

[117] Bangera M. K. , Kotian R. , and N R. , Effect of Titanium Dioxide Nanoparticle Reinforcement on Flexural Strength of Denture Base Resin: A Systematic Review and Meta-Analysis, Japanese Dental Science Review. (2020) 56, no. 1, 68–76, 10.1016/j.jdsr.2020.01.001. PMC7037538 32123548

[118] Imani M. M. , Kiani M. , Rezaei F. , Souri R. , and Safaei M. , Optimized Synthesis of Novel Hydroxyapatite/CuO/TiO_2_ Nanocomposite With High Antibacterial Activity Against Oral Pathogen *Streptococcus mutans* , Ceramics International. (2021) 47, no. 23, 33398–33404, 10.1016/j.ceramint.2021.08.246.

[119] Safaei M. and Taran M. , Optimal Conditions for Producing Bactericidal Sodium Hyaluronate-TiO_2_ Bionanocomposite and Its Characterization, International Journal of Biological Macromolecules. (2017) 104, 449–456, 10.1016/j.ijbiomac.2017.06.016. 28619641

[120] Nakano R. , Hara M. , and Ishiguro H. , et al.Broad Spectrum Microbicidal Activity of Photocatalysis by TiO_2_ , Catalysts. (2013) 3, no. 1, 310–323, 10.3390/catal3010310.

[121] Foster H. A. , Ditta I. B. , Varghese S. , and Steele A. , Photocatalytic Disinfection Using Titanium Dioxide: Spectrum and Mechanism of Antimicrobial Activity, Applied Microbiology and Biotechnology. (2011) 90, no. 6, 1847–1868, 10.1007/s00253-011-3213-7. PMC7079867 21523480

[122] Esteban Florez F. L. , Kraemer H. , and Hiers R. D. , et al.Sorption, Solubility and Cytotoxicity of Novel Antibacterial Nanofilled Dental Adhesive Resins, Scientific Reports. (2020) 10, no. 1, 10.1038/s41598-020-70487-z, 13503. PMC7421579 32782299

[123] Ahmad Fauzi N. A. , Ireland A. J. , Sherriff M. , Bandara H. M. H. N. , and Su B. , Nitrogen Doped Titanium Dioxide as an Aesthetic Antimicrobial Filler in Dental Polymers, Dental Materials. (2022) 38, no. 1, 147–157, 10.1016/j.dental.2021.10.019. 34836699

[124] Ansari S. A. , Khan M. M. , Ansari M. O. , and Cho M. H. , Nitrogen-Doped Titanium Dioxide (N-Doped TiO_2_) for Visible Light Photocatalysis, New Journal of Chemistry. (2016) 40, no. 4, 3000–3009, 10.1039/C5NJ03478G.

[125] Florez F. L. E. , Hiers R. D. , and Larson P. , et al.Antibacterial Dental Adhesive Resins Containing Nitrogen-Doped Titanium Dioxide Nanoparticles, Materials Science and Engineering C. (2018) 93, 931–943. 10.1016/j.msec.2018.08.060 PMC6500090 30274130

[126] Hiers R. D. , Huebner P. , Khajotia S. S. , and Florez F. L. E. , Characterization of Experimental Nanoparticulated Dental Adhesive Resins With Long-Term Antibacterial Properties, Nanomaterials. (2022) 12, no. 21, 10.3390/nano12213732, 3732. PMC9656602 36364508

[127] Zhang F. , Wu C. , and Zhou Z. , et al.Blue-Light-Activated Nano-TiO_2_@ PDA for Highly Effective and Nondestructive Tooth Whitening, ACS Biomaterials Science & Engineering. (2018) 4, no. 8, 3072–3077, 10.1021/acsbiomaterials.8b00548. 33435027

[128] Saberi A. , Baltatu M. S. , and Vizureanu P. , Recent Advances in Magnesium–magnesium Oxide Nanoparticle Composites for Biomedical Applications, Bioengineering. (2024) 11, no. 5, 10.3390/bioengineering11050508, 508. PMC11117911 38790374

[129] Shah R. , Khan D. , and Al-Anazi A. , et al.Non-Metal Doped ZnO and TiO_2_ Photocatalysts for Visible Light Active Degradation of Pharmaceuticals and Hydrogen Production: A Review, Applied Catalysis O: Open. (2025) 204, 10.1016/j.apcato.2025.207043, 207043.

[130] Giti R. , Zomorodian K. , Firouzmandi M. , Zareshahrabadi Z. , and Rahmannasab S. , Antimicrobial Activity of Thermocycled Polymethyl Methacrylate Resin Reinforced With Titanium Dioxide and Copper Oxide Nanoparticles, International Journal of Dentistry. (2021) 2021, no. 1, 8, 10.1155/2021/6690806, 6690806. PMC7868146 33603788

[131] Thakur N. , Thakur N. , and Kumar A. , et al.A Critical Review on the Recent Trends of Photocatalytic, Antibacterial, Antioxidant and Nanohybrid Applications of Anatase and Rutile TiO_2_ Nanoparticles, Science of the Total Environment. (2024) 914, 10.1016/j.scitotenv.2023.169815, 169815. 38184262

[132] Luttrell T. , Halpegamage S. , Tao J. , Kramer A. , Sutter E. , and Batzill M. , Why Is Anatase a Better Photocatalyst Than Rutile?-Model Studies on Epitaxial TiO_2_ Films, Scientific Reports. (2014) 4, no. 1, 10.1038/srep04043, 4043. PMC3918909 24509651

[133] Liu F. , Hong T. , Xie J. , Zhan X. , and Wang Y. , Application of Reactive Oxygen Species-Based Nanomaterials in Dentistry: A Review, Crystals. (2021) 11, no. 3, 10.3390/cryst11030266, 266.

[134] Kotta M. , Gorantla S. , and Muddada V. , et al.Antibacterial Activity and Debonding Force of Different Lingual Retainers Bonded With Conventional Composite and Nanoparticle Containing Composite: An In Vitro Study, Journal of the World Federation of Orthodontists. (2020) 9, no. 2, 80–85, 10.1016/j.ejwf.2020.03.001. 32672659

[135] Sodagar A. , Akhoundi M. S. A. , and Bahador A. , et al.Effect of TiO_2_ Nanoparticles Incorporation on Antibacterial Properties and Shear Bond Strength of Dental Composite Used in Orthodontics, Dental Press Journal of Orthodontics. (2017) 22, no. 5, 67–74, 10.1590/2177-6709.22.5.067-074.oar. PMC5730138 29160346

[136] Ren J. , Du Z. , and Lin J. , Applied Research Glass Ionomer Cement With TiO_2_ Nanoparticles in Orthodontic Treatment, Journal of Nanoscience and Nanotechnology. (2021) 21, no. 2, 1032–1041, 10.1166/jnn.2021.18681. 33183440

[137] Behnaz M. , Kasraei S. , Yadegari Z. , Zare F. , and Nahvi G. , Effects of Orthodontic Bonding Containing TiO_2_ and ZnO Nanoparticles on Prevention of White Spot Lesions: An In Vitro Study, Biointerface Research in Applied Chemistry. (2021) 12, no. 1, 431–440, 10.33263/BRIAC121.431440.

[138] Ghasemi T. , Arash V. , Rabiee S. M. , Rajabnia R. , Pourzare A. , and Rakhshan V. , Antimicrobial Effect, Frictional Resistance, and Surface Roughness of Stainless Steel Orthodontic Brackets Coated With Nanofilms of Silver and Titanium Oxide: A Preliminary Study, Microscopy Research and Technique. (2017) 80, no. 6, 599–607, 10.1002/jemt.22835. 28181353

[139] Ma Y. , Zhang N. , Weir M. D. , Bai Y. , and Xu H. H. K. , Novel Multifunctional Dental Cement to Prevent Enamel Demineralization Near Orthodontic Brackets, Journal of Dentistry. (2017) 64, 58–67, 10.1016/j.jdent.2017.06.004. 28642057

[140] Sharifian S. , Loghmani A. , Nayyerain S. , Javanbakht S. , and Daneii P. , Application of Magnesium Oxide Nanoparticles in Dentistry: A Literature Review, European Journal of General Dentistry. (2023) 12, no. 1, 1–6, 10.1055/s-0042-1760673.

[141] Vega-Jimenez A. L. , Gonzalez-Alva P. , Rodriguez-Hernandez A. P. , Vazquez-Olmos A. R. , and Paz-Diaz B. , Oxide Nanoparticles Based in Magnesium as a Potential Dental Tool to Inhibit Bacterial Activity and Promote Osteoblast Viability, Dental Materials Journal. (2024) 43, no. 1, 11–19, 10.4012/dmj.2023-041. 38072414

[142] Gatou M.-A. , Skylla E. , and Dourou P. , et al.Magnesium Oxide (MgO) Nanoparticles: Synthetic Strategies and Biomedical Applications, Crystals. (2024) 14, no. 3, 10.3390/cryst14030215, 215.

[143] Wang Y. , Wu Z. , and Wang T. , et al.Bioactive Dental Resin Composites With MgO Nanoparticles, ACS Biomaterials Science & Engineering. (2023) 9, no. 8, 4632–4645, 10.1021/acsbiomaterials.3c00490. 37486960

[144] Sideridou I. D. and Achilias D. S. , Elution Study of Unreacted Bis-GMA, TEGDMA, UDMA, and Bis-EMA From Light-Cured Dental Resins and Resin Composites Using HPLC, Journal of Biomedical Materials Research Part B: Applied Biomaterials. (2005) 74B, no. 1, 617–626, 10.1002/jbm.b.30252. 15889433

[145] Xue Q. , Wu Z. , and Wang W. , et al.Magnesium Oxide Nanoparticle-Enhanced Bioactive 3D-Printed Denture Resins: Optimized Antifungal Performance and Improved Clinical Applicability, Colloids and Surfaces A: Physicochemical and Engineering Aspects. (2025) 726, 10.1016/j.colsurfa.2025.137992, 137992.

[146] Rodríguez-Hernández A.-P. , Vega-Jiménez A. L. , Vázquez-Olmos A. R. , Ortega-Maldonado M. , and Ximenez-Fyvie L.-A. , Antibacterial Properties In Vitro of Magnesium Oxide Nanoparticles for Dental Applications, Nanomaterials. (2023) 13, no. 3, 10.3390/nano13030502, 502. PMC9921384 36770464

[147] Meran Z. D. , Hassan P. A. , and Salaie R. N. , Comparing the Antibacterial Effect of Coated and Impregnated Flexible Dentures With Magnesium Oxide Nanoparticles Against *streptococcus mutans* , Coatings. (2023) 13, no. 8, 10.3390/coatings13081429, 1429.

[148] Thamizharasan S. , Gurunathan K. , Varaprasad K. , and Chandrasekaran K. , Green Engineering of MgO Nanoparticles: Assessment of Their Antioxidant and Antibacterial Activity Against Dental Pathogens, Inorganic Chemistry Communications. (2024) 169, 10.1016/j.inoche.2024.113025, 113025.

[149] Noori A. J. and Kareem F. A. , The Effect of Magnesium Oxide Nanoparticles on the Antibacterial and Antibiofilm Properties of Glass-Ionomer Cement, Heliyon. (2019) 5, no. 10, 10.1016/j.heliyon.2019.e02568. PMC6812241 31667407

[150] Fan X. , Du J. , Li Y. , Duan K. , and Liu G. , Electrophoretic Deposition of Magnesium Oxide Coating on Micro-Arc Oxidized Titanium for Antibacterial Activity and Biocompatibility, Journal of Orthopaedic Surgery and Research. (2023) 18, no. 1, 10.1186/s13018-023-04390-4, 901. PMC10680288 38012792

[151] Yudovin-Farber I. , Beyth N. , Nyska A. , Weiss E. I. , Golenser J. , and Domb A. J. , Surface Characterization and Biocompatibility of Restorative Resin Containing Nanoparticles, Biomacromolecules. (2008) 9, no. 11, 3044–3050, 10.1021/bm8004897. 18821794

[152] Yudovin-Farber I. , Beyth N. , Weiss E. I. , and Domb A. J. , Antibacterial Effect of Composite Resins Containing Quaternary Ammonium Polyethyleneimine Nanoparticles, Journal of Nanoparticle Research. (2010) 12, no. 2, 591–603, 10.1007/s11051-009-9628-8.

[153] Beyth N. , Houri-Haddad Y. , Baraness-Hadar L. , Yudovin-Farber I. , Domb A. J. , and Weiss E. I. , Surface Antimicrobial Activity and Biocompatibility of Incorporated Polyethylenimine Nanoparticles, Biomaterials. (2008) 29, no. 31, 4157–4163, 10.1016/j.biomaterials.2008.07.003. 18678404

[154] Beyth N. , Pilo R. , and Weiss E. I. , Antibacterial Activity of Dental Cements Containing Quaternary Ammonium Polyethylenimine Nanoparticles, Journal of Nanomaterials. (2012) 2012, no. 1, 10.1155/2012/814763, 814763.

[155] Zaltsman N. , Kesler-Shvero D. , Weiss E. I. , and Beyth N. , Synthesis Variants of Quaternary Ammonium Polyethyleneimine Nanoparticles and Their Antibacterial Efficacy in Dental Materials, Journal of Applied Biomaterials & Functional Materials. (2016) 14, no. 2, 205–211, 10.5301/jabfm.5000269. 27032864

[156] Sharon E. , Sharabi R. , and Eden A. , et al.Antibacterial Activity of Orthodontic Cement Containing Quaternary Ammonium Polyethylenimine Nanoparticles Adjacent to Orthodontic Brackets, International Journal of Environmental Research and Public Health. (2018) 15, no. 4, 10.3390/ijerph15040606, 606. PMC5923648 29584643

[157] Zaltsman N. , Shvero D. K. , Polak D. , Weiss E. I. , Beyth N. , and Kesler Shvero D. , Antibacterial Orthodontic Adhesive Incorporating Polyethyleneimine Nanoparticles, Oral Health & Preventive Dentistry. (2017) 15, no. 3, 245–250, 10.3290/j.ohpd.a38525. 28674704

[158] Egorov A. R. , Kirichuk A. A. , Rubanik V. V. , Rubanik V. V.Jr, Tskhovrebov A. G. , and Kritchenkov A. S. , Chitosan and Its Derivatives: Preparation and Antibacterial Properties, Materials. (2023) 16, no. 18, 10.3390/ma16186076, 6076. PMC10532898 37763353

[159] Mirani S. A. , Sangi L. , Kumar N. , and Ali D. , Investigating the Antibacterial Effect of Chitosan in Dental Resin Composites: A Pilot Study, Pakistan Oral & Dental Journal. (2015) 35, no. 2.

[160] Dobrzyński W. , Piszko P. J. , and Kiryk J. , et al.Dental Resin Composites Modified With Chitosan: A Systematic Review, Marine Drugs. (2025) 23, no. 5, 10.3390/md23050199, 199. PMC12113295 40422789

[161] Kikuchi L. N. T. , Freitas S. R. M. , and Amorim A. F. , et al.Effects of the Crosslinking of Chitosan/DCPA Particles in the Antimicrobial and Mechanical Properties of Dental Restorative Composites, Dental Materials. (2022) 38, no. 9, 1482–1491, 10.1016/j.dental.2022.06.024. 35835609

[162] Elsaka S. E. , Antibacterial Activity and Adhesive Properties of a Chitosan-Containing Dental Adhesive, Quintessence International. (2012) 43, no. 7, 603–613. 22670256

[163] Dacoreggio R. , Bridi E. C. , and Basting R. T. , et al.Incorporation of Chitosan Into a Universal Adhesive System: Physicochemical Characteristics, Gelatinolytic Activity, Bond Strength and Interface Micromorphology Analyses, International Journal of Adhesion and Adhesives. (2021) 106, 10.1016/j.ijadhadh.2021.102814, 102814.

[164] Alves L. V. G. L. , Cerqueira N. M. , Candemil A. P. , Faria-e-Silva A. L. , Sousa-Neto M. D. , and Souza-Gabriel A. E. , Does Dentin Pretreatment With Chitosan Improve the Bond Strength of Restorative Material? a Systematic Review and Meta-Analysis of In Vitro Studies, International Journal of Adhesion and Adhesives. (2024) 128, 10.1016/j.ijadhadh.2023.103553, 103553.

[165] Ziotti I. R. , Paschoini V. L. , Corona S. A. M. , and Souza-Gabriel A. E. , Chitosan-Induced Biomodification on Demineralized Dentin to Improve the Adhesive Interface, Restorative Dentistry & Endodontics. (2022) 47, no. 3, 10.5395/rde.2022.47.e28. PMC9436653 36090512

[166] M El Azzazy A. , S Ali M. , and A Abdelrahim R. , The Effect of Chitosan Incorporation on Some Properties and Antibacterial Activity of Dental Adhesive, Al-Azhar Journal of Dental Science. (2018) 21, no. 4, 357–364, 10.21608/ajdsm.2018.71600.

[167] Divakar D. D. , Jastaniyah N. T. , Altamimi H. G. , Alnakhli Y. O. , Alkheraif A. A. , and Haleem S. , Enhanced Antimicrobial Activity of Naturally Derived Bioactive Molecule Chitosan Conjugated Silver Nanoparticle Against Dental Implant Pathogens, International Journal of Biological Macromolecules. (2018) 108, 790–797, 10.1016/j.ijbiomac.2017.10.166. 29102795

[168] Amin F. , Fareed M. A. , Zafar M. S. , Khurshid Z. , Palma P. J. , and Kumar N. , Degradation and Stabilization of Resin-Dentine Interfaces in Polymeric Dental Adhesives: An Updated Review, Coatings. (2022) 12, no. 8, 10.3390/coatings12081094, 1094.

[169] Zhang C. , Hui D. , and Du C. , et al.Preparation and Application of Chitosan Biomaterials in Dentistry, International Journal of Biological Macromolecules. (2021) 167, 1198–1210, 10.1016/j.ijbiomac.2020.11.073. 33202273

[170] Chandrasekaran M. , Kim K. , and Chun S. , Antibacterial Activity of Chitosan Nanoparticles: A Review, Processes. (2020) 8, no. 9, 10.3390/pr8091173, 1173.

[171] Al-hijazi A. Y. , Hasan N. , and Nasr B. K. , et al.Recent Advances in the use of Inorganic Nanomaterials as Anti Caries Agents, Heliyon. (2023) 9, no. 4, 10.1016/j.heliyon.2023.e15326. PMC10126947 37113794

[172] Javed R. , Rais F. , and Fatima H. , et al.Chitosan Encapsulated ZnO Nanocomposites: Fabrication, Characterization, and Functionalization of Bio-Dental Approaches, Materials Science and Engineering C. (2020) 116, 10.1016/j.msec.2020.111184, 111184. 32806262

[173] Lai C.-C. , Lin C.-P. , and Wang Y.-L. , Development of Antibacterial Composite Resin Containing Chitosan/Fluoride Microparticles as Pit and Fissure Sealant to Prevent Caries, Journal of Oral Microbiology. (2022) 14, no. 1, 10.1080/20002297.2021.2008615, 2008615. PMC8725701 34992735

[174] Ravandi R. , Heris S. Z. , Hemmati S. , Aghazadeh M. , Davaran S. , and Abdyazdani N. , Effects of Chitosan and TiO_2_ Nanoparticles on the Antibacterial Property and Ability to Self-Healing of Cracks and Retrieve Mechanical Characteristics of Dental Composites, Heliyon. (2024) 10, no. 6, 10.1016/j.heliyon.2024.e27734. PMC10957383 38524556

[175] Chatzistavrou X. , Lefkelidou A. , and Papadopoulou L. , et al.Bactericidal and Bioactive Dental Composites, Frontiers in Physiology. (2018) 9, 103. 10.3389/fphys.2018.00103 PMC5820345 29503619

[176] Faggiani A. and Falzon B. G. , Predicting Low-Velocity Impact Damage on a Stiffened Composite Panel, Composites Part A: Applied Science and Manufacturing. (2010) 41, no. 6, 737–749, 10.1016/j.compositesa.2010.02.005.

[177] Tanaka C. J. , Rodrigues J. A. , and Pingueiro J. M. S. , et al.Antibacterial Activity of a Bioactive Tooth-Coating Material Containing Surface Pre-Reacted Glass in a Complex Multispecies Subgingival Biofilm, Pharmaceutics. (2023) 15, no. 6, 10.3390/pharmaceutics15061727, 1727. PMC10302393 37376175

[178] Alreshidi S. , Evaluation of Ion Release, Antibacterial Characteristics, and Optical Properties of Newly Formulated Dental Composites With Zinc-Containing Glass Fillers, 2023.

[179] van de Lagemaat M. , Grotenhuis A. , and van de Belt-Gritter B. , et al.Comparison of Methods to Evaluate Bacterial Contact-Killing Materials, Acta Biomaterialia. (2017) 59, 139–147, 10.1016/j.actbio.2017.06.042. 28666886

[180] Cunliffe A. J. , Askew P. D. , and Stephan I. , et al.How Do We Determine the Efficacy of an Antibacterial Surface? a Review of Standardised Antibacterial Material Testing Methods, Antibiotics. (2021) 10, no. 9, 10.3390/antibiotics10091069, 1069. PMC8472414 34572650

[181] Camilleri J. , Moliz T. A. , and Bettencourt A. , et al.Standardization of Antimicrobial Testing of Dental Devices, Dental Materials. (2020) 36, no. 3, e59–e73, 10.1016/j.dental.2019.12.006. 31928776

[182] Consul S. , An Evaluation of Antibacterial Activity of Three Different Root Repair Materials Using Agar-Diffusion Test and Direct Contact Test an In-Vitro Study, 2016, Rajiv Gandhi University of Health Sciences (India).

[183] Ampah-Essel J. E. , Barszczewska-Rybarek I. , Drejka P. , and Chladek G. , Quaternary Ammonium Dimethacrylates as an Additive in Dental Composite Resins: A Review of Their Antimicrobial, Mechanical, and Physicochemical Properties, Materials. (2025) 18, no. 21, 10.3390/ma18214844, 4844. PMC12610268 41227801

[184] Wilson C. , Lukowicz R. , and Merchant S. , et al.Quantitative and Qualitative Assessment Methods for Biofilm Growth: A Mini-Review, Research & Reviews. Journal of Engineering and Technology. (2017) 6, no. 4. PMC6133255 30214915

[185] Welch K. , Cai Y. , and Strømme M. , A Method for Quantitative Determination of Biofilm Viability, Journal of Functional Biomaterials. (2012) 3, no. 2, 418–431, 10.3390/jfb3020418. PMC4047939 24955541

[186] Sinha A. , Mallick U. , Singh S. , Mishra P. R. , Sahu M. C. , and Panda S. K. , Effective Methods of Bacterial Biofilm Measurements, Bacterial Biofilm and Chronic Infections: Role in Disease Pathogenesis and Therapeutic Strategies, 2025, Springer, 285–315.

[187] Negrea Ş.M. , Methods for Quantifying and Characterizing Bacterial Biofilms, Romanian Archives of Microbiology and Immunology. (2020) 79, no. 2, 122–132.

[188] Hanif A. and Ghani F. , Mechanical Properties of an Experimental Resin Based Composite Containing Silver Nanoparticles and Bioactive Glass, Pakistan Journal of Medical Sciences. (2020) 36, no. 4, 10.12669/pjms.36.4.1913, 776. PMC7260929 32494273

[189] Brandão N. L. , Portela M. B. , Maia L. C. , Antônio A. , Silva V. L. M. , and da Silva E. M. , Model Resin Composites Incorporating ZnO-NP: Activity Against *S. mutans* and Physicochemical Properties Characterization, Journal of Applied Oral Science. (2018) 26, 10.1590/1678-7757-2017-0270. PMC5933836 29742262

[190] Almukhlifi H. , Effectiveness of Se/ZnO NPs in Enhancing the Antibacterial Activity of Resin-Based Dental Composites, Materials. (2022) 15, no. 21, 10.3390/ma15217827. PMC9658905 36363419

[191] Shoorgashti R. , Shahrzad H. , Nowroozi S. , Ghadamgahi B. , Mehrara R. , and Oroojalian F. , Evaluation of the Antibacterial and Cytotoxic Activities of Ag/ZnO Nanoparticles Loaded Polycaprolactone/Chitosan Composites for Dental Applications, Nanomedicine Journal. (2023) 10, no. 1.

[192] Mirhashemi A. H. , Bahador A. , Kassaee M. Z. , Daryakenari G. , Ahmad-Akhoundi M. S. , and Sodagar A. , Antimicrobial Effect of Nano-Zinc Oxide and Nano-Chitosan Particles in Dental Composite Used in Orthodontics, Journal of Medical Bacteriology. (2013) 2, no. 3-4, 1–10.

[193] Ali S. , Sangi L. , Kumar N. , Kumar B. , Khurshid Z. , and Zafar M. S. , Evaluating Antibacterial and Surface Mechanical Properties of Chitosan Modified Dental Resin Composites, Technology and Health Care. (2020) 28, no. 2, 165–173, 10.3233/THC-181568. 31594266

[194] Jafari S. , Mahyad B. , and Hashemzadeh H. , et al.Biomedical Applications of Titanium Dioxide (TiO_2_) Nanoparticles, Phytopharmacology Research Journal. (2024) 3, no. 3, 29–40.

[195] Esteban Florez F. L. , Trofimov A. A. , Ievlev A. , Qian S. , Rondinone A. J. , and Khajotia S. S. , Advanced Characterization of Surface-Modified Nanoparticles and Nanofilled Antibacterial Dental Adhesive Resins, Scientific Reports. (2020) 10, no. 1, 10.1038/s41598-020-66819-8, 9811. PMC7299952 32555360

[196] Kong X. , Han Q. , and Jiang A. , et al.BNN/TiO_2_ Nanocomposite System–modified Dental Flow Resins and the Mechanism of the Enhancement of Mechanical and Antibacterial Properties, Biomaterials Science. (2023) 11, no. 8, 2775–2786, 10.1039/D2BM01848A. 36825578

[197] Sharifianjazi F. , Sharifianjazi M. , Irandoost M. , Tavamaishvili K. , Mohabatkhah M. , and Montazerian M. , Advances in Zinc-Containing Bioactive Glasses: A Comprehensive Review, Journal of Functional Biomaterials. (2024) 15, no. 9, 10.3390/jfb15090258, 258. PMC11433484 39330233

[198] Muhammad Syazwan M. N. and Yanny Marliana B. I. , The Influence of Simultaneous Divalent Cations (Mg^2+^, Co^2+^ and Sr^2+^) Substitution on the Physico-Chemical Properties of Carbonated Hydroxyapatite, Ceramics International. (2019) 45, no. 12, 14783–14788, 10.1016/j.ceramint.2019.04.208.

[199] Naruphontjirakul P. , Kanchanadumkerng P. , and Ruenraroengsak P. , Multifunctional Zn and Ag Co-Doped Bioactive Glass Nanoparticles for Bone Therapeutic and Regeneration, Scientific Reports. (2023) 13, no. 1, 10.1038/s41598-023-34042-w, 6775. PMC10130135 37185618

[200] Cao X. , Jia J. , and Wu L. , et al.Antibacterial and Metalloproteinase-Inhibited Zinc-Doped Bioactive Glass Nanoparticles for Enhancing Dentin Adhesion, Journal of Dentistry. (2025) 154, 10.1016/j.jdent.2025.105610, 105610. 39909140

[201] Zhou Y. , Hiraishi N. , Shimada Y. , Wang G. , Tagami J. , and Feng X. , Evaluation of Tooth Demineralization and Interfacial Bacterial Penetration Around Resin Composites Containing Surface Pre-Reacted Glass-Ionomer (S-PRG) Filler, Dental Materials. (2021) 37, no. 5, 849–862, 10.1016/j.dental.2021.02.009. 33674077

[202] Nedeljkovic I. , De Munck J. , and Vanloy A. , et al.Secondary Caries: Prevalence, Characteristics, and Approach, Clinical Oral Investigations. (2020) 24, no. 2, 683–691, 10.1007/s00784-019-02894-0. 31123872

[203] Cazzaniga G. , Ottobelli M. , and Ionescu A. C. , et al.In Vitro Biofilm Formation on Resin-Based Composites After Different Finishing and Polishing Procedures, Journal of Dentistry. (2017) 67, 43–52, 10.1016/j.jdent.2017.07.012. 28750776

[204] Kuang X. , Chen V. , and Xu X. , Novel Approaches to the Control of Oral Microbial Biofilms, BioMed Research International. (2018) 2018, no. 1, 13, 10.1155/2018/6498932, 6498932. PMC6330817 30687755

[205] Kumar B. , Kashyap N. , Avinash A. , Chevvuri R. , Sagar M. K. , and Shrikant K. , The Composition, Function and Role of Saliva in Maintaining Oral Health: A Review, Proteins-Structure Function and Bioinformatics. (2017) 220, 140–640.

[206] Hannig M. and Joiner A. , The Structure, Function and Properties of the Acquired Pellicle, Monographs in Oral Science. (2005) 19, 29–64, 10.1159/000090585. 16374028

[207] Marsh P. D. and Zaura E. , Dental Biofilm: Ecological Interactions in Health and Disease, Journal of Clinical Periodontology. (2017) 44, no. S18, S12–S22, 10.1111/jcpe.12679. 28266111

[208] Sang T. , Ye Z. , and Fischer N. G. , et al.Physical-Chemical Interactions Between Dental Materials Surface, Salivary Pellicle and *Streptococcus gordonii* , Colloids and Surfaces B: Biointerfaces. (2020) 190, 10.1016/j.colsurfb.2020.110938, 110938. PMC7440396 32172164

[209] Salli K. M. and Ouwehand A. C. , The use of *In Vitro* Model Systems to Study Dental Biofilms Associated With Caries: A Short Review, Journal of Oral Microbiology. (2015) 7, no. 1, 10.3402/jom.v7.26149, 26149. PMC4349908 25740099

[210] Abuljadayel R. , Aljadani N. , Almutairi H. , and Turkistani A. , Effect of Antibacterial Agents on Dentin Bond Strength of Bioactive Restorative Materials, Polymers. (2023) 15, no. 12, 10.3390/polym15122612, 2612. PMC10300955 37376257

[211] Jones J. R. , Review of Bioactive Glass: From Hench to Hybrids, Acta Biomaterialia. (2013) 9, no. 1, 4457–4486, 10.1016/j.actbio.2012.08.023. 22922331

[212] Cury J. A. and Tenuta L. M. A. , Enamel Remineralization: Controlling the Caries Disease or Treating Early Caries Lesions?, Brazilian Oral Research. (2009) 23, no. suppl 1, 23–30, 10.1590/S1806-83242009000500005. 19838555

[213] Kumar V. V. , Harnany D. , and Dzanzani R. , et al.DLP-Printed Bio-Based Mini-Channel Filters for Water Filtration: Influence of Channel Size and Surface Quality on Mechanical and Filtration Performance, Materials Technology. (2026) 41, no. 1, 10.1080/10667857.2026.2630055, 2630055.

[214] Alshali R. Z. , Salim N. A. , Satterthwaite J. D. , and Silikas N. , Long-Term Sorption and Solubility of Bulk-Fill and Conventional Resin-Composites in Water and Artificial Saliva, Journal of Dentistry. (2015) 43, no. 12, 1511–1518, 10.1016/j.jdent.2015.10.001. 26455541

[215] Kunrath M. F. and Dahlin C. , The Impact of Early Saliva Interaction on Dental Implants and Biomaterials for Oral Regeneration: An Overview, International Journal of Molecular Sciences. (2022) 23, no. 4, 10.3390/ijms23042024, 2004. PMC8875286 35216139

[216] Jafer M. A. , Qadiri A. A. , Mtwam N. A. , Hakami A. H. , and Mowkly A. A. , Influence of Human and Bacterial Enzymes on Resin Restorations: A Review, The Journal of Contemporary Dental Practice. (2022) 23, no. 3, 371–377, 10.5005/jp-journals-10024-3250. 35781444

[217] Guo X. , Yu Y. , Gao S. , Zhang Z. , and Zhao H. , Biodegradation of Dental Resin-Based Composite—A Potential Factor Affecting the Bonding Effect: A Narrative Review, Biomedicines. (2022) 10, no. 9, 10.3390/biomedicines10092313, 2313. PMC9496159 36140414

[218] Bourbia M. and Finer Y. , Biochemical Stability and Interactions of Dental Resin Composites and Adhesives With Host and Bacteria in the Oral Cavity: A Review, Journal (Canadian Dental Association). (2018) 84, i1. 29513214

[219] Finer Y. and Santerre J. P. , Salivary Esterase Activity and Its Association With the Biodegradation of Dental Composites, Journal of Dental Research. (2004) 83, no. 1, 22–26, 10.1177/154405910408300105. 14691108

[220] Abdelaziz A. A. , Doghish A. S. , and Salah A. N. , et al.When Oral Health Affects Overall Health: Biofilms, Dental Infections, and Emerging Antimicrobial Strategies, Infection. (2025) 53, no. 5, 1603–1624, 10.1007/s15010-025-02533-9. 40261483

[221] Kumar V. V. , Rajendran S. , Surendran S. , and Ramakrishna S. , Enhancing the Properties of Carbon Fiber Thermoplastic Composite by Nanofiber Interleaving, *2022 IEEE International Conference on Nanoelectronics, Nanophotonics, Nanomaterials, Nanobioscience & Nanotechnology (5NANO)*, 2022, IEEE, 1–4.

[222] Vijay Kumar V. , Balaganesan G. , Lee J. K. Y. , Neisiany R. E. , Surendran S. , and Ramakrishna S. , A Review of Recent Advances in Nanoengineered Polymer Composites, Polymers. (2019) 11, no. 4, 10.3390/polym11040644, 644. PMC6523580 30970621

[223] S S. , R G A. P. , and Bajaj G. , et al.A Review on the Recent Applications of Synthetic Biopolymers in 3D Printing for Biomedical Applications, Journal of Materials Science: Materials in Medicine. (2023) 34, no. 12, 1–22, 10.1007/s10856-023-06765-9. PMC10661719 37982917

[224] Kumar V. V. , Katamneni P. , Chandran S. , and Muflikhun M. A. , Failure of Lightweight Composites: A Machine Learning Approach, Lightweight Composites, 2026, Elsevier, 231–266.

[225] Zhou X. , Huang X. , and Li M. , et al.Development and Status of Resin Composite as Dental Restorative Materials, Journal of Applied Polymer Science. (2019) 136, no. 44, 10.1002/app.48180, 48180.

[226] Kumar V. V. , Narayanan D. , Chandran S. , Rajendran S. , and Ramakrishna S. , Lightweight and Sustainable Self-Reinforced Composites, Lightweight and Sustainable Composite Materials, 2023, Elsevier, 19–46.

[227] Srivastava A. A. , Bondarev V. P. , and Kostyleva E. I. , et al.Doped TiO_2_ and Oligoalkylhydrosiloxane-Based Surface Modifiers With Self-Cleaning and Hydrophobic Properties, Next Materials. (2026) 10, 10.1016/j.nxmate.2025.101414, 101414.

[228] Correia C. O. , Leite Á.J. , and Mano J. F. , Chitosan/Bioactive Glass Nanoparticles Scaffolds With Shape Memory Properties, Carbohydrate Polymers. (2015) 123, 39–45, 10.1016/j.carbpol.2014.12.076. 25843832

[229] Sergi R. , Bellucci D. , and Cannillo V. , A Review of Bioactive Glass/Natural Polymer Composites: State of the Art, Materials. (2020) 13, no. 23, 10.3390/ma13235560, 5560. PMC7730917 33291305

[230] Couto D. S. , Hong Z. , and Mano J. F. , Development of Bioactive and Biodegradable Chitosan-Based Injectable Systems Containing Bioactive Glass Nanoparticles, Acta Biomaterialia. (2009) 5, no. 1, 115–123, 10.1016/j.actbio.2008.08.006. 18835230

[231] Xing Z. , Zhu L. , and Wu Y. , et al.Effect of Nano-TiO_2_ Particle Size on the Bonding Performance and Film-Forming Properties of Starch-Based Wood Adhesives, International Journal of Biological Macromolecules. (2023) 235, 10.1016/j.ijbiomac.2023.123697, 123697. 36806780

[232] Usman M. S. , El Zowalaty M. E. , Shameli K. , Zainuddin N. , Salama M. , and Ibrahim N. A. , Synthesis, Characterization, and Antimicrobial Properties of Copper Nanoparticles, International Journal of Nanomedicine. (2013) 8, 4467–4479, 10.2147/IJN.S50837. PMC3839804 24293998

[233] Jafari A. , Pourakbar L. , Farhadi K. , Mohamadgolizad L. , and Goosta Y. , Biological Synthesis of Silver Nanoparticles and Evaluation of Antibacterial and Antifungal Properties of Silver and Copper Nanoparticles, Turkish Journal of Biology. (2015) 39, no. 4, 556–561, 10.3906/biy-1406-81.

[234] Veloso R. C. , Maia J. , and Praça R. , et al.Impact of SiO_2_, TiO_2_ and ZnO Nanoparticles Incorporation on the Thermo-Optical Properties of Dark-Coloured Façade Coatings, Journal of Building Engineering. (2024) 84, 10.1016/j.jobe.2024.108517, 108517.

[235] AL-Rubaiawi H. , Alwan A. F. , Husain A. A. , Ibrahim N. M. , Ali D. A. K. , and Mahmood A. T. , Structural and Optical Properties of Doped Polystyrene Thin Films by (NiO, TiO_2_, ZnO, MgO) Nanoparticles, Progress in Color, Colorants and Coatings. (2025) 18, no. 3, 323–341.

[236] Šupová M. , Martynková G. S. , and Barabaszová K. , Effect of Nanofillers Dispersion in Polymer Matrices: A Review, Science of Advanced Materials. (2011) 3, no. 1, 1–25, 10.1166/sam.2011.1136.

[237] Vijayan M. , Selladurai V. , and Kumar V. V. , Investigating the Influence of Nano-Silica on Low-Velocity Impact Behavior of Aluminium-Glass Fiber Sandwich Laminate, Silicon. (2023) 15, no. 11, 4845–4859, 10.1007/s12633-023-02391-w.

[238] Arun D. , Adikari Mudiyanselage D. , Gulam Mohamed R. , Liddell M. , Monsur Hassan N. M. , and Sharma D. , Does the Addition of Zinc Oxide Nanoparticles Improve the Antibacterial Properties of Direct Dental Composite Resins? A Systematic Review, Materials. (2020) 14, no. 1, 10.3390/ma14010040, 40. PMC7795203 33374229

[239] Beyth N. , Yudovin-Farber I. , Basu A. , Weiss E. I. , and Domb A. J. , Antimicrobial Nanoparticles in Restorative Composites, Emerging Nanotechnologies in Dentistry, 2018, Elsevier, 41–58, 10.1016/B978-0-12-812291-4.00003-0.

[240] Elfakhri F. , Alkahtani R. , Li C. , and Khaliq J. , Influence of Filler Characteristics on the Performance of Dental Composites: A Comprehensive Review, Ceramics International. (2022) 48, no. 19, 27280–27294, 10.1016/j.ceramint.2022.06.314.

[241] Habib E. , Wang R. , Wang Y. , Zhu M. , and Zhu X. X. , Inorganic Fillers for Dental Resin Composites: Present and Future, ACS Biomaterials Science & Engineering. (2016) 2, no. 1, 1–11, 10.1021/acsbiomaterials.5b00401. 33418639

[242] Sreena R. , Raman G. , Manivasagam G. , and Nathanael A. J. , Bioactive Glass–polymer Nanocomposites: A Comprehensive Review on Unveiling Their Biomedical Applications, Journal of Materials Chemistry B. (2024) 12, no. 44, 11278–11301, 10.1039/D4TB01525H. 39392456

[243] Masoumi M. , Valizadeh S. , Carvalho R. M. , Moghaddam A. A. , and Ghodsi S. , The Effect of Adding Chitosan Nanoparticles on Different Properties of the Adhesive and High-Filled Composite Resin, International Journal of Adhesion and Adhesives. (2024) 134, 10.1016/j.ijadhadh.2024.103766, 103766.

[244] Miresmaeili A. , Atai M. , Mansouri K. , and Farhadian N. , Effect of Nanosilver Incorporation on Antibacterial Properties and Bracket Bond Strength of Composite Resin, Iranian Journal of Orthodontics. (2012) 7, no. 3, 14–19.

[245] Afkhami F. , Elahy S. , and Mahmoudi-Nahavandi A. , Spectrophotometric Analysis of Crown Discoloration Following the use of Silver Nanoparticles Combined With Calcium Hydroxide as Intracanal Medicament, Journal of Clinical and Experimental Dentistry. (2017) 9, no. 7, 10.4317/jced.53743. PMC5549580 28828148

[246] Al-Dulaijan Y. A. , AlGhamdi M. A. , Azmy E. , Al-Kholy M. R. Z. , Almulhim K. S. , and Helal M. A. , Color Stability of Nanoparticles-Modified Dental Resin-Based Composites, Applied Sciences. (2023) 13, no. 6, 10.3390/app13063870, 3870.

[247] Safitri R. N. , Suriani A. B. , and Htwe Y. Z. N. , et al.Recent Development of Electrochemically Exfoliated Graphene and Its Hybrid Conductive Inks for Printed Electronics Applications, Synthetic Metals. (2024) 308, 10.1016/j.synthmet.2024.117707, 117707.

[248] Wais U. , Jackson A. W. , He T. , and Zhang H. , Nanoformulation and Encapsulation Approaches for Poorly Water-Soluble Drug Nanoparticles, Nanoscale. (2016) 8, no. 4, 1746–1769, 10.1039/C5NR07161E. 26731460

[249] Emam A.-N. M. , Ahmed K.-E. I. , Shaaban A. M. , Alqhtani M. A. , Gad M. M. , and Helal M. A. , Color and Flexural Properties of Nanoparticles-Modified Denture Base Resin: An In Vitro Comparative Study, International Journal of Health Sciences. (2024) 18, no. 3, 23–29. PMC11075442 38721136

[250] Meher R. , Mallick R. R. , Sarangi P. , Jena A. , Suman S. , and Sharma G. , Optimization of Chitosan Nanoparticle Dentin Pretreatment With Different Concentrations and Application Times to Improve Bonding at Resin-Dentin Interface, Journal of Conservative Dentistry and Endodontics. (2025) 28, no. 3, 248–252, 10.4103/JCDE.JCDE_850_24. PMC12007738 40256700

[251] Tanpichai S. , Yuwawech K. , Wimolmala E. , Srimarut Y. , Woraprayote W. , and Malila Y. , Enhanced Physical, Mechanical and Barrier Properties of Chitosan Films via Tannic Acid Cross-Linking, RSC Advances. (2025) 15, no. 37, 30742–30757, 10.1039/D5RA04227E. PMC12395003 40895732

[252] Carrouel F. , Viennot S. , Ottolenghi L. , Gaillard C. , and Bourgeois D. , Nanoparticles as Anti-Microbial, Anti-Inflammatory, and Remineralizing Agents in Oral Care Cosmetics: A Review of the Current Situation, Nanomaterials. (2020) 10, no. 1, 10.3390/nano10010140, 140. PMC7022934 31941021

[253] Spinola M. S. , Moecke S. E. , Rossi N. R. , Nakatsuka T. , Borges A. B. , and Torres C. R. G. , Efficacy of S-PRG Filler Containing Varnishes on Enamel Demineralization Prevention, Scientific Reports. (2020) 10, no. 1, 10.1038/s41598-020-76127-w, 18992. PMC7642416 33149256

[254] Ge Y. , Wang S. , Zhou X. , Wang H. , Xu H. , and Cheng L. , The use of Quaternary Ammonium to Combat Dental Caries, Materials. (2015) 8, no. 6, 3532–3549, 10.3390/ma8063532. PMC4665981 26635932

[255] Iordache C. , Fătu A. M. , Ancuța C. , Bogdan V. , and Antohe M. E. , Oral Syndrome in Some Nutrition Disorders, Diabetes-Ergonomical Approaches, Romanian Journal of Oral Rehabilitation. (2020) 12, no. 1.

[256] Molania T. , Alimohammadi M. , Akha O. , Mousavi J. , Razvini R. , and Salehi M. , The Effect of Xerostomia and Hyposalivation on the Quality of Life of Patients With Type II Diabetes Mellitus, Electronic Physician. (2017) 9, no. 11, 5814–5819, 10.19082/5814. PMC5783133 29403624

[257] Ali A. M. M. , Jabir S. M. , Kadhim A. A. , and Almagtome A. , Nanotechnology Practices and Cost Restructure for Effective Cost Management Under Industry 4.0 Based Manufacturing Systems, TEM Journal. (2022) 11, no. 3.

[258] Kumar R. , Thakur A. , Hajam Y. A. , Bala I. , and Singh S. , Diagnostic Techniques Related to the Oral Microbiome, Oral Microbiome. (2025) 346–374, 10.1201/9781003478638-19.

